# Theranostic approach to specifically targeting the interloop region of *BCL2* i-motif DNA by crystal violet

**DOI:** 10.1038/s41598-023-39407-9

**Published:** 2023-09-01

**Authors:** Sinjan Das, Shuntaro Takahashi, Tatsuya Ohyama, Sudipta Bhowmik, Naoki Sugimoto

**Affiliations:** 1 Frontier Institute for Biomolecular Engineering Research (FIBER), Konan University, 7-1-20 Minatojima-Minamimachi, Kobe, 650-0047 Japan; 2 Department of Biophysics, Molecular Biology and Bioinformatics, University of Calcutta, 92, A.P.C Road, Kolkata, 700009 India; 3 Mahatma Gandhi Medical Advanced Research Institute (MGMARI), Sri Balaji Vidyapeeth (Deemed to be University), Pondy-Cuddalore Main Road, Pillayarkuppam, Pondicherry, 607402 India; 4 Graduate School of Frontiers of Innovative Research in Science and Technology (FIRST), Konan University, 7-1-20 Minatojima-Minamimachi, Kobe, 650-0047 Japan

**Keywords:** Biochemistry, Biophysical chemistry, DNA

## Abstract

Ligands that recognise specific i-motif DNAs are helpful in cancer diagnostics and therapeutics, as i-motif formation can cause cancer. Although the loop regions of i-motifs are promising targets for ligands, the interaction between a ligand and the loop regions based on sequence information remains unexplored. Herein, we investigated the loop regions of various i-motif DNAs to determine whether these regions specifically interact with fluorescent ligands. Crystal violet (CV), a triphenylmethane dye, exhibited strong fluorescence with the i-motif derived from the promoter region of the human *BCL2* gene in a sequence- and structure-specific manner. Our systematic sequence analysis indicated that CV was bound to the site formed by the first and third loops through inter-loop interactions between the guanine bases present in these loops. As the structural stability of the *BCL2* i-motif was unaffected by CV, the local stabilisation of the loops by CV could inhibit the interaction of transcription factors with these loops, repressing the *BCL2* expression of MCF-7 cells. Our finding suggests that the loops of the i-motif can act as a novel platform for the specific binding of small molecules; thus, they could be utilised for the theranostics of diseases associated with i-motif DNAs.

## Introduction

Nucleic acids preserve genetic information to form canonical duplexes with Watson–Crick base pairings. Alternatively, non-canonical structures exist as other structures via non-Watson–Crick base pairs, such as triplexes and tetraplexes, including the guanine quadruplex (G4) and i-motif^1–3^. I-motif is one of the tetraplexes formed in cytosine (C) -rich sequences and comprises a pair of parallel duplexes with intercalating hemi-protonated C:C^+^ base pairs^4^. As the p*K*_a_ value of deoxycytidine is about 4.3, i-motif formation is usually favoured in mildly acidic conditions, although some i-motif structures can exist at neutral pH^5, 6^. I-motif formations are also stabilised by molecular crowding conditions^7–10^. Because the intracellular state is highly crowded with biomolecules, metabolites^11^, i-motif stability is higher in cells than in vitro^12, 13^. Moreover, the i-motif formations in human cells depend on their cell cycles, suggesting that i-motifs might have regulatory roles in cells^14^. The potential i-motif forming sequences are frequently found within gene promoter regions^15^. Some i-motifs on cancer and neurodegenerative disease genes, such as *BCL2*, *KRAS*, *HRAS*, and *VEGF,* were identified to regulate their gene expressions^16–19^. Therefore, detecting and regulating a certain i-motif in various crowding conditions is necessary for the theranostics of cancer and neurodegenerative diseases.

The most promising approach for detecting and regulating i-motifs involves proteins or small molecules that specifically bind to a certain i-motif. Although there are several proteins that bind to C-rich sequences^20–23^, they do not specifically recognise i-motif DNAs over other nucleic acid structures. Therefore, the antibody for i-motifs, iMab, is the only protein capable of binding to i-motif structures, and can detect various i-motifs with high specificity and affinity over other nucleic acid structures^14^. The use of antibodies for i-motif detection is valuable in identifying the formation of i-motif DNAs under different intracellular conditions, such as changes in cell-cycle and pH. However, this approach is limited to visualising i-motifs without distinguishing a specific i-motif, as demonstrated by iMab's recognition capability for the i-motif scaffold, regardless of its sequence variations^14^. Instead, the small molecules can be utilised to detect i-motifs in living cells, and various ligands that bind to i-motifs were also reported^24, 25^. However, there are only a few specific ligands for certain i-motifs, such as IMC-48 for the *BCL2* i-motif^16^, NSC309874 for the *PDGFR-β* i-motif^26^, and an acridone derivative B19 for the *c-MYC* i-motif^27^. Detection and regulation of i-motif formation by fisetin, which binds to a hairpin-like structure converted from the *VEGF* i-motif to emit fluorescence and regulate replication efficiency, was reported only by our group^28^. To develop a specific i-motif ligand for theranostic application, the ligand must generally recognise the unique structure of the i-motif and other motifs by fluorescence emission. The i-motif core is a common scaffold of C:C^+^ base pairs, while the loop regions connecting the core are specific for each sequence. In the loop regions, the nucleobases can interact with each other^29^, creating a specific site for ligand binding^30^. Thus, a ligand that recognises both core and loop regions should be specific for a certain i-motif. In case of G4s, specific small ligands that discriminate G4 topologies have been reported, which may interact directly with G4 loops and grooves^31–35^. To facilitate the development of theranostics for a specific i-motif, a platform ligand is required to design a dual-targeting ligand binding to both the core and loops of i-motifs.

To find the platform ligand, we focused on the ligands that exhibit very low fluorescence response in the absence of DNA but reveal significant enhancement in emission intensity upon binding with DNA. The ligand we require should interact with both the core and loop region of i-motifs. Triphenylmethane dyes, which possess propeller structure and various dialkyl amino substitutions could be useful due to their low fluorescence emission in water^36^. In this study, we explored the loop regions of different i-motifs and studied their interactions with different triphenylmethane dyes. Among the triphenylmethane dyes, crystal violet (CV) showed the highest fluorescence response with the i-motif from the promoter region of the human *BCL2* gene, compared to that of other structures, such as other i-motifs, G4s, and hairpin. We found that CV bound to the site involving the first and third loops of the *BCL2* i-motif, where the guanine bases in the loops contributed to the specific binding of CV. The complexation of the loop regions and CV repressed *BCL2* gene transcription in the human breast cancer cell line of MCF-7. Our approach suggests that the loop region of i-motif can serve as a novel framework for the specific binding of small molecules and can potentially develop novel ligands to monitor and regulate the biological reactions of i-motif DNAs.

## Results and discussion

### Design of different i-motif-forming DNA sequences to screen the specific binding of triphenylmethane derivatives

**Figure 1:**
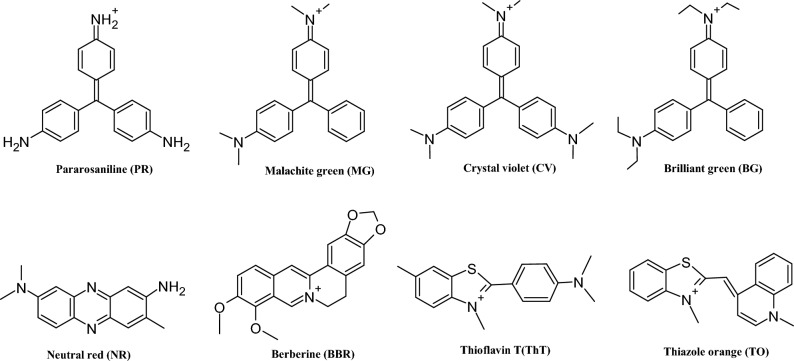
Chemical structures of the fluorescent ligands used in this study.

As the region of the edge of the i-motif core is smaller than that of G4s, the ligand should be smaller than the size of a G-quartet and have multiple binding motifs to interact with both the core of the i-motif C:C^+^ base pair and loop regions. Therefore, triphenylmethane molecules such as pararosaniline (PR), malachite green (MG), CV, and brilliant green (BG) were chosen (Fig. 1). These derivatives have a positively charged amino group which possibly interacts with the negatively charged phosphate backbone of DNA. Furthermore, different alkyl substitutions on the amino groups also facilitate DNA binding through favourable van der Waals interactions. These molecules are known to bind to nucleic acids, including i-motif to emit fluorescence, although the fluorescence is not high^37^. For reference, several i-motif binding ligands, such as neutral red (NR)^38^, berberine (BBR)^39^, thioflavin T (ThT)^40^, and thiazole orange (TO)^41^ were also tested. For DNA sequences, i-motifs are usually categorised into class I motifs having loop bases less than 4 and class II motifs having loop bases greater than 4^42^. Here, we investigated natural forming i-motif sequences of *C9ORF72*, human telomere region (hTelo), *HRAS*, *ILPR*, and *VEGF* genes from class I type and *BCL2*, *HIF-1α*, *JAZF1*, mid region of *KRAS* (*KRAS* mid), and *PDGF-A* genes from class II type (Table 1). The formation of i-motifs and/or roles in the gene expression of these sequences were identified in living human cells^3, 13^. In addition to these natural sequences, some additional i-motif sequences of class II having C_3_-tracts with varying numbers of only thymidine in loops from five to eight were also tested (Table 1). The control sequences were chosen from G4 DNA sequences having different topologies (parallel; *BCL2* G4, *MYC* G4 and *VEGF* G4, hybrid; human telomere G4, and anti-parallel; thrombin binding aptamer (TBA)), and hairpin DNA having a 9 mers stem (Table 1). All sequences formed i-motifs, G4s with different topologies, and hairpins, respectively, as expected in the solution containing 10 mM KH_2_PO_4_, 50 mM KCl, and 1 mM K_2_EDTA with pH 5.0 at 25 °C (Fig. S1).

**Table 1:** DNA sequences used in this study (5ʹ–3ʹ).

Name	Category	Sequence
*C9ORF72* iM	Class I i-motif	GGCCCCGGCCCCGGCCCCGGCCCC
hTelo iM	Class I i-motif	CCCTAACCCTAACCCTAACCCTAA
*HRAS* iM1	Class I i-motif	CCCCCGCCCCCGCCCCGCCCCG
*HRAS* iM2	Class I i-motif	CCCCCGCCCCCGCCCCGCCCCGGCCTCG
*ILPR* iM	Class I i-motif	CCCCACACCCCTGTCCCCACACCCC
*VEGF* iM	Class I i-motif	GACCCCGCCCCCGGCCCGCCCCGG
*BCL2* iM	Class II i-motif	CAGCCCCGCTCCCGCCCCCTTCCTCCCGCGCCCGCCCCT
*HIF-1α* iM^a^	Class II i-motif	CGCGCTCCCGCCCCCTCTCCCCTCCCCGCGC
*JAZF1* iM^a^	Class II i-motif	CCCCCCCCGCCCCCGCCCCCGCCCTCCCCCC
*KRAS* mid iM	Class II i-motif	GCCCGGCCCCCGCTCCTCCCCCGCCGGCCCGGCCCGGCCCCCTCCTTCTCCCCG
*KRAS* mid I-IV iM	Class II i-motif	GCCCGGCCCCCGCTCCTCCCCCGCCGGCCCGG
*PDGF-A* iM	Class II i-motif	CCGCGCCCCTCCCCCCGCCCCCCGCCCCCCGCCCCCCCCCCCCC
C3T555 iM	Class II i-motif	CCCTTTTTCCCTTTTTCCCTTTTTCCC
C3T666 iM	Class II i-motif	CCCTTTTTTCCCTTTTTTCCCTTTTTTCCC
C3T777 iM	Class II i-motif	CCCTTTTTTTCCCTTTTTTTCCCTTTTTTTCCC
C3T888 iM	Class II i-motif	CCCTTTTTTTTCCCTTTTTTTTCCCTTTTTTTTCCC
*BCL2* G4	Parallel G4	AGGGGCGGGCGCGGGAGGAAGGGGGCGGGAGCGGGGCTG
hTelo G4	Hybrid G4	AGGGTTAGGGTTAGGGTTAGGG
*MYC* G4	Parallel G4	TGAGGGTGGGTAGGGTGGGTAA
TBA G4	Anti-parallel G4	GGTTGGTGTGGTTGG
*VEGF* G4	Parallel G4	GGGGCGGGCCGGGGGCGGGG
Hairpin (H2)	Hairpin	CTATCGGACTTCGGTCCGATAG
H3	G-rich non-G4	AGGGTTAGGGTTAGGGTT
R1	Random sequence	TTGCCTTGCTGCTCTACCTCCA
C29 iM	Reference sequence	CCCCCTTTCCCCCTTTCCCCCTTTCCCCC
CV30S	Reference sequence	AACGACCACCGGTGCGCCGTACAGGTAACTAGCGTCGTCGTT

^a^Although we are considering them as class II i-motifs, the potential arrangement of the loop regions of the *HIF-1α* and *JAZF1* i-motifs is not available. Therefore, they can be considered as class I i-motifs depending on the loop arrangements^5^.

### Specific fluorescence emission upon binding of crystal violet to *BCL2* iM

**Figure 2:**
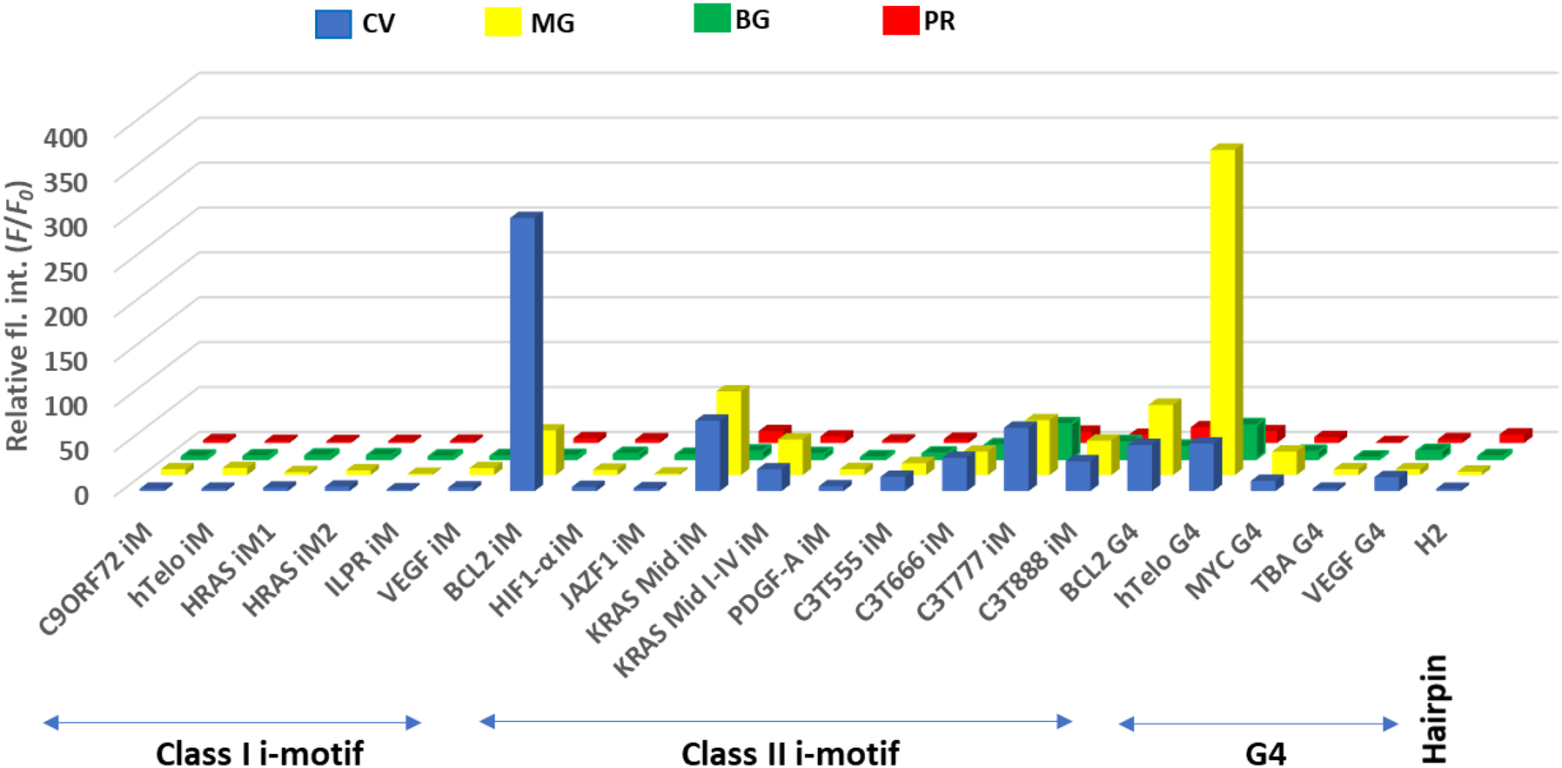
Fluorescence responses of CV (blue), MG (yellow), BG (green) and PR (red) with different DNA motifs. The data was relative to no DNA as the control. The errors were within 5%.

**Figure 3:**
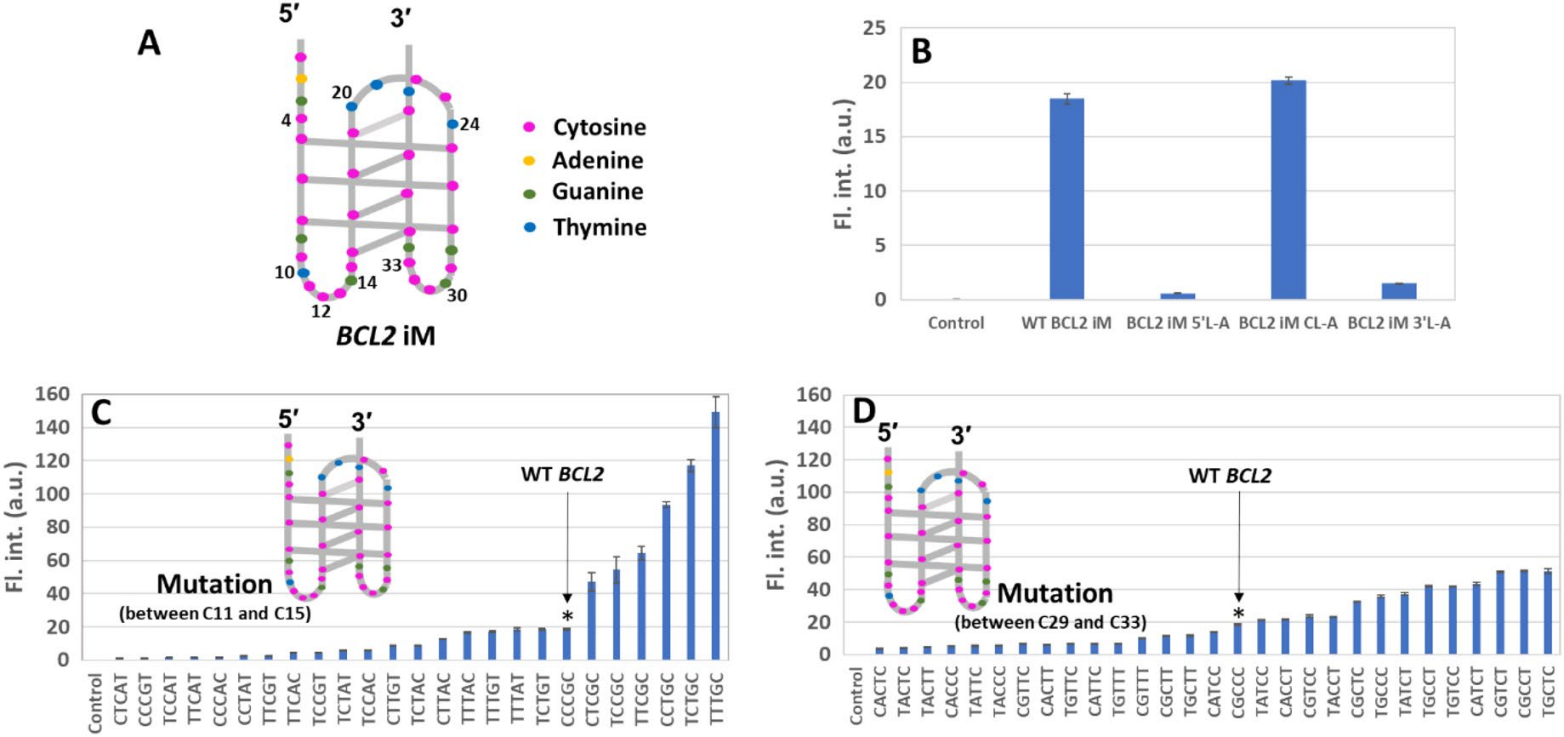
(**A**) Schematic illustration of the *BCL2* iM structure. Fluorescence responses of CV with (**B**) the wild type (WT) *BCL2* iM sequence and its abasic loop sequences in the 5′ loop (5′L-A), central loop (CL-A), and 3′ loop (3′L-A). (**C**) Systematic mutations in the 5′ loop, and (**D**) systematic mutations in the 3′ loop. CV in only buffer was used as the control.

The fluorescence response of triphenylmethane derivatives in the presence of different i-motifs (shorter loop: class I, and longer loop: class II), G4s, and hairpin DNA sequences was tested at pH 5.0 using 6.0 µM of each ligand with 15 µM of DNA at 25 °C to ensure 1:1 ligand-DNA binding. In the presence of CV, class I i-motifs showed a slight increase in fluorescence (Fig. 2), and the fluorescence level of CV (*F*/*F*_0_; *F* = fluorescence intensity of CV in the presence of DNA and *F*_0_ = fluorescence intensity of CV without DNA) with these sequences were less than 5, whereas a significant fluorescence increase was observed in the presence of the *BCL2* i-motif DNA (*BCL2* iM), a class II i-motif (Fig. 2). It has been identified that *BCL2* iM has three long loops (5′ loop; GCTCCCGC, central loop; TTCCT and 3′ loop; GCGCCCG, Fig. 3A)^16, 43, 44^. The *F*/*F*_0_ of CV was about 300-fold with *BCL2* iM. Compared with the *BCL2* iM, the fluorescence level with other sequences was less significant. In the Class II group, *KRAS* mid iM, and its core C-rich region (*KRAS* mid I–IV iM) exhibited relatively high fluorescence (*F*/*F*_0_ = 78.5 and 24.3, respectively). The model sequences also showed relatively high fluorescence and among them, C3T777 iM showed the largest response (*F*/*F*_0_ = 70.5). However, other class II i-motifs (*HIF1-α*, *JAZF1*, and *PDGF-A*) showed negligible fluorescence (*F*/*F*_0_ values were below 6). These results indicate that both the length and sequences of loop regions of the i-motif had an important role in CV binding and its fluorescence emission. For G4 structures, the fluorescence level of CV with hTelo G4 (*F*/*F*_0_ = 53.0) and *BCL2* G4 (*F*/*F*_0_ = 51.4) were relatively high, although the fluorescence enhancement was much smaller than that with *BCL2* iM (*F*/*F*_0_ = 304.3), demonstrating higher selectivity of CV for *BCL2* iM compared with the selectivity of CV for hTelo G4, as we previously reported^45^. Moreover, the hairpin DNA exhibited almost no change in fluorescence level. The *BCL2* iM fluorescence was 130-fold higher than that of the hairpin DNA. Previously, C29 iM, a model i-motif sequence having five C:C^+^ base pairs and the loops containing three thymidines exhibited CV fluorescence^37^ and a specific aptamer, CV30S, for CV was developed (Table 1)^46^. In both the cases, the *F*/*F*_0_ values were 2.0 and 18.2 (Fig. S2), revealing much lower fluorescence enhancement when compared to that of *BCL2* iM. Extending the fluorescence assay experiment to a neutral pH condition (pH  7.0 at 25 °C), we observed a significant reduction in the fluorescence of CV in the presence of i-motif forming sequences, particularly with *BCL2* iM (Fig. S3). However, no significant change was observed with G4 and hairpin forming sequences, indicating that the fluorescence properties of CV is negligibly affected by the pH of the medium. Therefore, the fluorescence experiment in a neutral pH environment demonstrates that the specific recognition of *BCL2* iM by CV is primarily determined by the secondary structure of the DNA rather than its primary sequence. To further demonstrate the significance of DNA secondary structures, we investigated the fluorescence response of CV in the presence of 50 mM LiCl instead of KCl at pH 8.0 at 25 °C. Under these conditions, iM and G4 forming DNA cannot form tetraplexes, and thus they serve as unstructured controls for C-rich non-iM forming DNA and G-rich non-G4 DNA, respectively. The fluorescence response of CV upon binding to the *BCL2* iM and G-rich non-G4 sequence (H3, Table 1) significantly decreased at pH 8.0 at 25 °C in the presence of LiCl, as compared to the results shown in Fig. 2 (Fig. S4). Moreover, CV did not exhibit a noteworthy fluorescence response (Fig. S4) in the presence of the randomly composed 22-mer DNA (R1, Table 1) under the same conditions. Thus, we established the significance of the DNA secondary structure in determining the fluorescence response of CV.

To understand the importance of the structural aspect of CV for its specificity towards *BCL2* iM, we checked the fluorescence responses of its derivatives, such as MG, BG, and PR, (Fig. 2) having the same core structure of triphenylmethane but different dialkylamino substitutions. A significant decrease in the relative fluorescence response of the MG-*BCL2* iM (*F*/*F*_0_ = 49.7) system relative to the CV-*BCL2* iM (*F*/*F*_0_ = 304.3) system, implies that the lack of one dimethyl amino group in MG compared with CV is responsible for the lowered fluorescence response. Besides *BCL2* iM, MG revealed considerable fluorescence light-up for *KRAS* mid iM (*F*/*F*_0_ = 93.3) and C3777 iM (*F*/*F*_0_ = 61.1) belonging to the class II i-motif category. For class I i-motifs, the fluorescence response of MG was very low (*F*/*F*_0_ < 8). Among the G4s, the relative fluorescence response of MG was maximum for hTelo G4 (*F*/*F*_0_ = 362.4), projecting MG as a specific probe for hTelo G4, which is already established^47^. In the case of BG, *F*/*F*_0_ for *BCL2* iM was 3.3, which was significantly lower than that of CV. The steric hindrance owing to the presence of two bulky diethyl amino groups in BG could be responsible for the low fluorescence response of BG towards *BCL2* iM as well as other i-motifs. PR containing three aniline groups showed low and comparable fluorescence response towards all DNA motifs studied here. These results indicate that CV interacted with *BCL2* iM in a structure-specific manner through all its three dimethyl amino groups, and emitted high fluorescence.

To confirm whether the fluorescence of CV upon binding to *BCL2* iM was due to the specificity of the ligand’s structure, we also studied the fluorescence responses of four known i-motif binding ligands NR, BBR, ThT, and TO (Fig. S5) in the presence of our studied library of different DNA motifs in pH 5.0 buffer solution at 25 °C. In the case of NR, almost no fluorescence enhancement was observed in the presence of *BCL2* iM (Fig. S5A). However, in the presence of *KRAS* mid iM, *F*/*F*_0_ of NR was about 20, although those for other natural class II sequences showed only about 2.5 or less. Among the model class II sequences, C3T777 iM showed the highest enhancement (*F*/*F*_0_ = 9.3). However, this fluorescence enhancement was much smaller than that of CV and *BCL2* iM. Other ligands did not show clear sequence specificity for fluorescence emission (Fig. S5B–D). Hence, compared with conventional i-motif binders, CV was specific for *BCL2* iM, implying its sequence-specific binding nature.

### Proposed binding mechanism of CV with *BCL2* iM

To investigate how CV is bound to the *BCL2* iM, we analysed the binding property of CV with *BCL2* iM. Job’s plot analysis revealed that CV formed a 1:1 complex with the i-motif (Fig. S6A). Ensuring 1:1 complexation, the binding constant of CV with *BCL2* iM was 8.0 × 10^4^ M^−1^ at 25 °C from fluorescence titration (Fig. S6B). The binding constant of CV with *BCL2* iM was smaller than the reported binding constant of the same with C29 iM (1.2 × 10^6^ M^−1^)^37^. Using molecular modelling, Ma et al. demonstrated that the probable binding mode of CV with C29 iM consisting of a short loop region (TTT) was through end-stacking to C:C^+^ base pairs, resulting in a high binding constant. However, unfavourable entropy contribution (see the following section) due to the interaction of CV with the long loop regions might be responsible for the lowered binding constant value.

Next, we replaced the sequence of the loop regions of *BCL2* iM and compared the fluorescence intensity of CV with specific replacements in the *BCL2* iM to study whether CV is bound to the loop region. The replacement of the nucleotides of the first and third loops at the 5ʹ or 3ʹ end of the i-motif with the abasic nucleotides (*BCL2* iM 5ʹL-A or *BCL2* iM 3ʹL-A, Table S1) significantly decreased the fluorescence intensity of CV when (Fig. 3B) compared to that of the wild type (WT) *BCL2* iM. However, the replacement with abasic oligonucleotides at the second loop of the central position (*BCL2* iM CL-A, Table S1) still showed a similar level as that of the WT *BCL2* iM. These results suggest that a single CV molecule bound to the region formed with the first and third loops through a sequence-specific manner.

**Figure 4:**
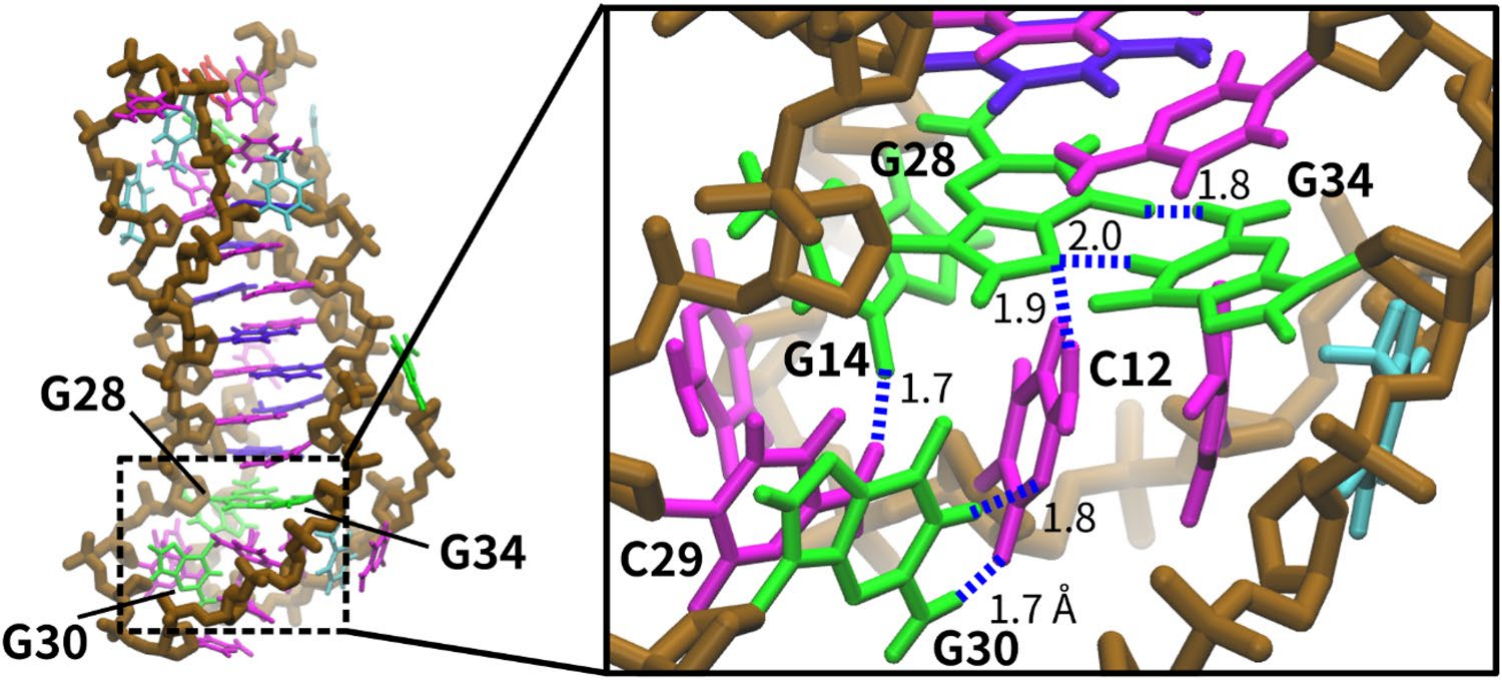
Representative structure of *BCL2* iM in the MD simulation. The panel on the right is the enlarged view of the loop-loop interactions. Representation of green, magenta, purple, cyan, and brown sticks show guanine, cytosine, protonated cytosine, and thymine bases, and the backbone, respectively. Blue lines represent the hydrogen bonds.

Considering the binding manner studied by these assays, the tertiary structure of the loop regions was studied by molecular dynamics (MD) simulations. Because the tertiary structure of *BCL2* iM has not been determined through NMR or X-ray crystallography, we utilised the solved structure of the human telomere i-motif as the initial model^48^. We used simulation data obtained during 100 ns simulation of three different runs and analysed the root mean square deviation (RMSD) calculated by fitting the coordinates of the C4’ atom in the core region of the C:C^+^ base pair and the first and third loop regions’ bases. RMSD data suggest that the core regions fluctuated less compared to the first and third loop regions (Fig. S7), because the loop regions should be more flexible than the core region. However, at the RMSD level, the first and third loop regions were in equilibrium, suggesting that the interactions form a preferred structure within the loop region. After 86 ns simulation, we sampled a snapshot of the simulated structure and found that there were inter-loop interactions between C12 in the first loop and G30 in the third loop (Fig. 4), forming a Watson–Crick-like base pair with two hydrogen bonds between them on the non-planar configuration. Other snapshots indicated interactions between C15 and G30 (Fig. S8). Therefore, it can be inferred that the first and third loops can interact through the cytosine bases in the first loop and G30 in the third loop. Furthermore, the MD simulation revealed that G14 in the first loop interacted with either C29 or C31 in the third loop. Moreover, G28 and G34 formed non-Watson–Crick base pairing (Fig. 4). These results suggest that the loop bases G14, G28, and G34 likely play a crucial role in creating the binding site for CV.

To confirm the abovementioned possibility, we systematically mutated the CCCGC sequence between C11 and C15 in the first loop and the CGCCC sequence between C29 and C33 in the third loop of the *BCL2* iM. Mutating each nucleotide individually will generate a large set of mutant sequences, which will pose difficulty in their analysis. Therefore, to decrease the number of mutated sequences, we chose to mutate C to T or G to A (Table S1) to generate a significant number of mutated sequences from which we could identify the loop nucleotides responsible for the specific binding sites of CV. Using CD spectra, we confirmed the folding of mutant sequences into an i-motif structure. Some of the representative CD spectra are shown in Fig. S1. Next, we checked the fluorescence response of CV with the mutated sequences (Fig. 3C, D). As expected, G14A mutation on the first loop significantly reduced fluorescence, indicating its crucial role in recognising CV by G14 (Fig. 3C). The magnitude of the emission intensity of CV with the 5′L-CCCAC mutant was tenfold lower than that of the WT, similar to that observed with the abasic sequences of the first loop (*BCL2* iM 5ʹL-A). In contrast, mutation at C11, C12, and C13 on the first loop to thymine (5′L-TTTGC) drastically increased the fluorescence, revealing the highest intensity. The magnitude of the intensity of CV with the 5′L-TTTGC mutant was eightfold higher than that of the WT. The binding constant of CV with the 5′L-TTTGC mutant (2.6 × 10^5^ M^−1^) was also higher than that with the WT *BCL2* iM at 25 °C (8.0 × 10^4^ M^−1^) (Fig. S6B). On the other hand, the 5′L-CTCAT mutant, which exhibited low fluorescence intensity, did not show significant increase in fluorescence intensity of CV (Fig. S6B). As the MD simulation data indicated that the cytosines could interact with G30 on the third loop, these interactions might sterically interfere with CV binding. The snapshot data of the MD simulation indicated that the 5′L-TTTGC mutant had less interaction between the first and third loops; thus, there was space for CV to easily access the binding site (Fig. S9). In the case of the mutation at the third loop, the fluorescence intensity was varied, but there was no clear sequence dependency on the magnitude of the fluorescence changes (Fig. 3D). Moreover, a ninefold decrease in the fluorescence response of CV due to the G34A mutation (Fig. S10) revealed the importance of G34, positioned as the nearest neighbour at the end of the C:C^+^ core, as a key nucleotide for creating a binding site of CV. Similarly, G28 can be considered as another key nucleotide, as evidenced from the MD simulation study. These results reveal that the specific bases of the first and third loops of *BCL2* iM form inter-loop hydrogen bonding and CV possibly interacts with these hydrogen-bonded loop structures through stacking and van der Waals interactions leading to enhanced fluorescence intensity of CV. Therefore, the dynamicity of the *BCL2* iM loops is a significant factor for CV fluorescence emission.

### Dynamic function of *BCL2* loops depending on the stability of i-motif with CV

To investigate how the stability of i-motif affects the fluorescence behaviour of CV, we measured the stability dependency of the *BCL2* iM on the fluorescence property. The UV melting analysis of *BCL2* iM was carried out at pH 5.0 in the same buffer condition as that in fluorescence assays (Fig. S11). The decrease in the absorbance at 295 nm in the presence of CV reveals the probable interaction between the loop region and CV. Both in the absence and presence of CV, the stability of *BCL2* iM at 37 °C (−∆*G*°_37_) at pH 5.0 with CV was both 6.1 kcal mol^−1^ (Table 2), indicating that *BCL2 * iM stability remains unaffected by CV. The change in the enthalpy (∆*H*°) and entropy parameters (*T*∆*S*°) for the i-motif formation did not reveal a significant change in the absence and presence of CV. Moreover, the melting temperatures (*T*_m_) of *BCL2* iM were found to be 65.4 °C both in the absence and presence of CV, indicating no overall stabilisation due to CV binding. We also performed the UV-melting experiments on the 5′L-TTTGC mutant in the absence and presence of CV (Fig. S11). A significant decrease in the absorbance at 295 nm revealed that the probable interaction between the loop region of the mutant sequence and CV was much higher compared to that with the WT sequence. However, the value of −∆*G*°_37_ of the 5′L-TTTGC mutant was 4.7 kcal mol^−1^ (Table 2), revealing a decrease in the i-motif stability of 1.4 kcal mol^−1^, compared with that of the WT *BCL2* iM. The *T*_m_ of the 5′L-TTTGC mutant was found to be 60.5 °C, which is 4.9 °C lower than that of the WT sequence. The decrease of 6.0 kcal mol^−1^ in enthalpy contribution [∆∆*H*° = (−∆*H*° for 5′L-TTTGC mutant) ‒ (−∆*H*° for WT *BCL2* iM)] for the 5′L-TTTGC mutant compared with the WT sequence could be responsible for the destabilisation. This destabilisation may be due to reduced interaction between the first and third loops than that in the WT *BCL2* iM, as shown in the MD simulation. However, in the presence of CV, the 5′L-TTTGC mutant showed significantly increased stability (−∆*G*°_37_ = 5.5 kcal mol^−1^), resulting in 0.8 kcal mol^−1^ stabilisation compared to that without CV (−∆*G*°_37_ = 4.7 kcal mol^−1^), which is also supported by a 1.1 °C increase in *T*_m_. ∆∆*H*°_L_ [∆∆*H*°_L_ = (−∆*H*° for 5′L-TTTGC mutant with CV) ‒ (−∆*H*° for 5′L-TTTGC mutant without CV)] and [∆(*T*∆*S*°_L_) = (−*T*∆*S*° for 5′L-TTTGC mutant with CV) ‒ (−*T*∆*S*° for 5′L-TTTGC mutant without CV)] values were 8.3 kcal mol^−1^ and 7.5 kcal mol^−1^, respectively, indicating that the binding of CV with the 5′L-TTTGC mutant is enthalpically favoured. This favourable enthalpy contribution could arise from an increased stacking interaction between the phenyl groups of CV with the loop bases of the 5′L-TTTGC mutant. These findings suggest that in the case of the WT sequence, the stabilisation effect on i-motif structure by CV binding and 0.8 kcal mol^−1^ stabilisation in the 5′L-TTTGC mutant, were cancelled out by the destabilisation of the loop-loop interaction formed before CV binding. On the other hand, the *−∆G*°_37_ of the 5′L-CTCAT mutant, which exhibited low fluorescence intensity, was found to be 4.7 kcal mol^−1^ in the absence of CV and 4.6 kcal mol^−1^ in the presence of CV (Table 2). Both values were very similar to that of the 5′L-TTTGC mutant in the absence of CV. Although the addition of CV did not stabilise the overall i-motif, the binding of CV led to the reorientation of the loop bases, indicating the flexibility of the loop region to generate specific binding sites for small molecules for increase in the affinity. Thus, even if the stability of the whole i-motif structure was not changed, the dynamics of the i-motif structure might be varied upon binding CV to the *BCL2* iM.

**Table 2:** Thermodynamic parameters and melting temperatures of *BCL2* iM^a^.

Sequence	pH	Ligand	−∆*H*° (kcal mol^−1^)	−*T*∆*S*^°^ (kcal mol^−1^)	−∆*G*°_37_ (kcal mol^−1^)	*T*_m_ (°C)
*BCL2* iM	5.0	No ligand	72.6 ± 1.6	66.5 ± 1.6	6.1 ± 0.2	65.4 ± 0.1
	5.0	CV	72.6 ± 0.4	66.5 ± 0.4	6.1 ± 0.3	65.4 ± 0.1
5′L-TTTGC	5.0 5.0	No ligand CV	66.6 ± 0.3 74.9 ± 1.6	61.9 ± 0.4 69.4 ± 1.6	4.7 ± 0.2 5.5 ± 0.2	60.5 ± 0.1 61.6 ± 0.2
5′L-CTCAT	5.0 5.0	No ligand CV	68.1 ± 1.2 68.5 ± 1.5	63.4 ± 0.5 63.9 ± 1.4	4.7 ± 0.2 4.6 ± 0.3	60.0 ± 0.1 59.4 ± 0.2
*BCL2* 2323	5.0	No ligand	55.0 ± 1.8	51.6 ± 1.7	3.4 ± 0.3	57.4 ± 0.2
*BCL2* 4545	5.0	No ligand	82.9 ± 2.5	74.6 ± 2.4	8.3 ± 0.5	71.5 ± 0.1
*BCL2* 5656	5.0	No ligand	77.3 ± 2.6	68.4 ± 2.5	8.9 ± 0.4	77.4 ± 0.2

^a^Buffer contained 10 mM KH_2_PO_4_, 1 mM K_2_EDTA and 50 mM KCl. The pH of the solution was maintained at 25 °C.

To understand the effect of CV binding on the dynamics of the i-motif structure, we tested different numbers of C-tracts of *BCL2* iM at pH 5.0 (Fig. S12A). The detailed study of Hurley’s group on the WT *BCL2* iM structure^43^ revealed that among the six consecutive cytosine runs (excluding C22 and C23), 3 C from run I (C5 to C7), 4 C from run III (C16 to C19), 3 C from run IV (C25 to C27), and 4 C from run VI (C35 to C38) participate in the formation of seven intercalated C:C^+^ base pairs, which we denote as C_3_C_4_C_3_C_4_ tracts. Extending this logic, we prepared *BCL2* 2323, 4545, and 5656 as artificial sequences to investigate the effect of C-tract on the loop region. It is well-established that the stability of the i-motif increases with an increasing number of C-tracts. Compared with the original sequence (WT *BCL*2 having C_3_C_4_C_3_C_4_ tracts), the *BCL2* 2323 sequence having C_2_C_3_C_2_C_3_ tracts exhibited reduced fluorescence intensity of CV, whereas *BCL2* 4545 (C_4_C_5_C_4_C_5_ tracts) and *BCL2* 5656 (C_5_C_6_C_5_C_6_ tracts) showed higher fluorescence intensities. The UV melting data indicated the stability increased depending on the C-tract number (Table 2), as expected^49^. However, all the melting profiles (Fig. S12B) indicated that the i-motifs were completely formed at 25 °C, which was the temperature at which fluorescence was measured. Thus, the core region stability might affect the loop regions dynamics, which regulate the loop function to accommodate CV for fluorescence emission. Our MD simulation revealed that G14, G28, and G34, which played key roles in creating binding sites for CV, were located at the nearest neighbour position of the end of the C:C^+^ core. The CV fluorescence, depending on the stability of the core, indicated that the dynamics of the core region propagated to the loop regions, resulting in the fluctuation of the binding site of CV followed by the different fluorescence emissions. These results suggest that the factors like solution properties, which alter the loop dynamics, can affect the fluorescence property of the CV-*BCL2* iM system.

### Characteristics of the CV-*BCL2* iM complex under molecular crowding

**Figure 5:**
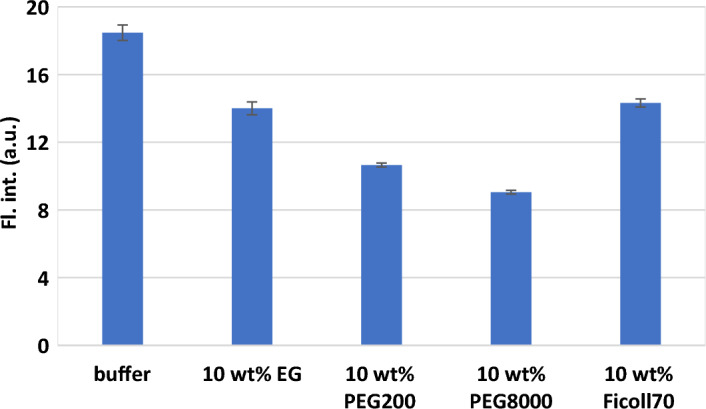
Fluorescence responses of CV with *BCL2* iM in the presence of different cosolutes.

Molecular crowding affects the physicochemical properties of the solution through excluded volume effect, alteration of water activity, dielectric constant, and medium viscosity. Molecular crowding can stabilise i-motif DNAs under various pH conditions including neutral pH due to the increase in the p*K*a value of cytosines^9, 13, 50^. However, limited information is available regarding the effect of molecular crowding on the loops of iMs. Therefore, to investigate the effect of molecular crowding with different solution properties on the dynamics of the loop region, the CV fluorescence with *BCL2* iM was investigated using various crowders. Here, we used cosolutes of different molecular weights, such as ethylene glycol (EG), PEG200, PEG8000, and Ficoll70. These different crowders can be utilised to mimic different intracellular crowding conditions. Previous reports have demonstrated that the solution containing different crowders, namely Ficoll70, PEG200, and BSA, can effectively mimic local intracellular conditions such as the nucleus, nucleolus, and cytosol, respectively^51^. Small cosolutes, such as EG and PEG200, effectively decrease a solution’s water activity and dielectric constant, whereas large cosolutes, such as PEG8000 and Ficoll70 cause the excluded volume effect. The fluorescence intensities of CV with *BCL2* iM remarkably decreased in 10 wt% EG and PEG200 solutions (Fig. 5) compared with that in the no cosolute condition. From UV melting assays (Fig. S13), the −∆*G*°_37_ values indicated that EG minimally destabilised *BCL2* iM, but no effective stabilisation effect was observed for PEG200 (Table S2), which were also supported by the *T*_m_ values of the i-motif in those respective solutions. Modulation of hydration state through uptake of water molecules by EG destabilised the i-motif structure^10^, lowering the fluorescence intensity. Both EG and PEG200 reduce water activity, thereby inhibiting hydration around DNA duplexes in the presence of these crowders^52^. Therefore, the presence of a long and ordered loop region of *BCL2* iM, which can serve as a scaffold for water molecules, may become destabilised in the presence of PEG200, balancing the core region stabilisation by the release of water molecules induced by the crowder^10^. Destabilisation of base pairings of loop regions in PEG200 was responsible for a larger decrease in the fluorescence intensity. Furthermore, Ficoll70 having only excluded volume effect showed a slight decrease in fluorescence signal. This result suggests that both the core and the loop structures might be stabilised by the excluded volume, but the energetic contribution between the core and the loop regions compensates for the effect on the stability of the i-motif and binding of CV. In the case of PEG8000, it was unexpected that the fluorescence signal decreased more than in EG- and PEG200. Previously, PEGs (of large molecular weights) were reported to directly interact with G4 to stabilise the structures^53^. Thus, it is possible that PEG8000 interacted with the guanines that acted as a structural platform for CV binding and repressed the CV binding on the loop regions. Except for PEG8000, the solution environment can indirectly and dynamically regulate the CV binding on *BCL2* iM. Furthermore, as we observed the hypochromisity of absorbance upon the melting assays in the presence of CV, all crowding agents did not cause major structural changes of the *BCL2* iM tetraplex (Fig. S13). Therefore, the crowding agents affected the structure and/or dynamics of loop regions. Hence, the CV binding in cells can be applied to detect and regulate the formation of *BCL2* iM depending on intracellular conditions.

### Effect of CV on *BCL2* gene expression in cells

**Figure 6:**
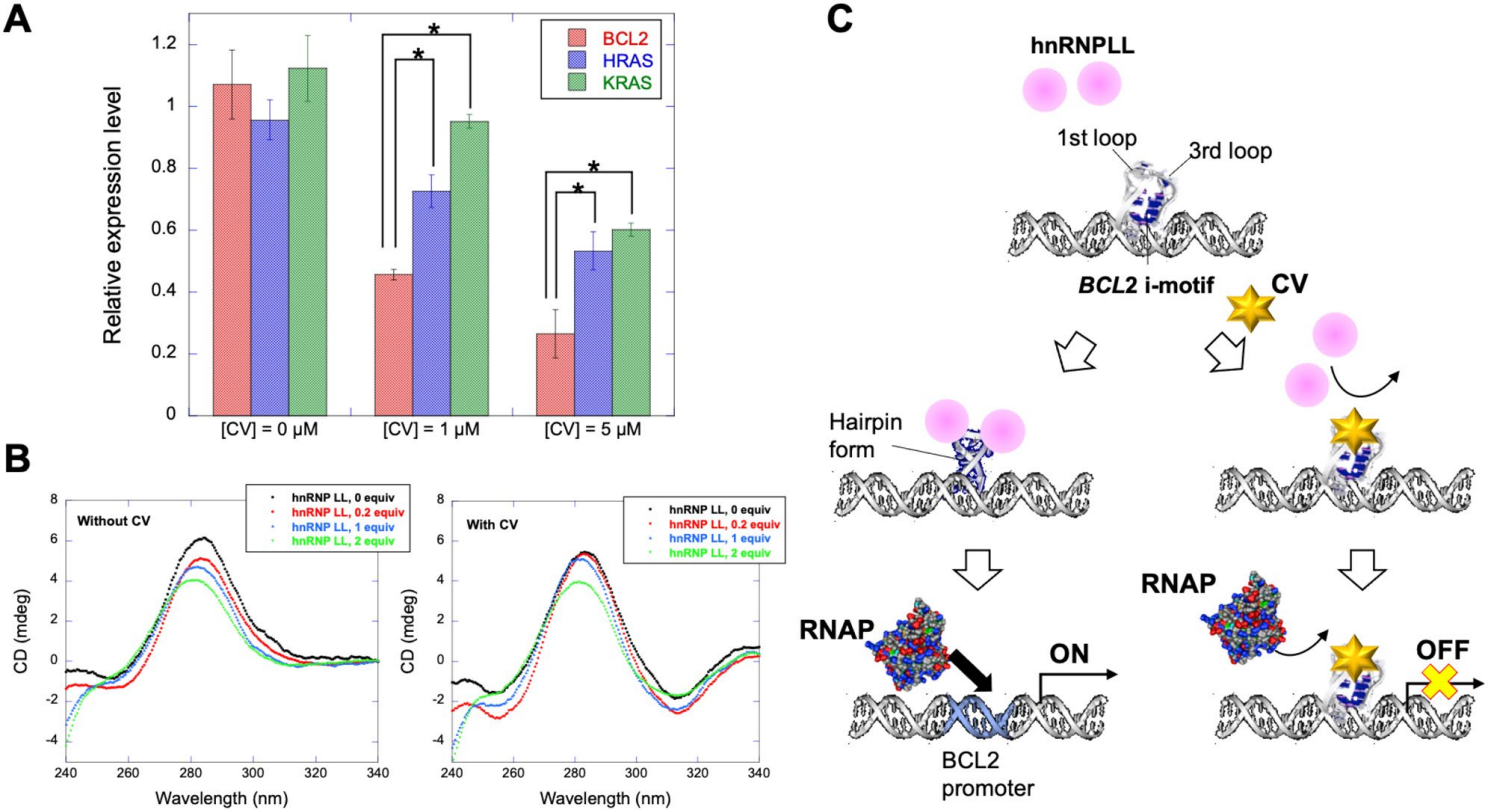
(**A**) Relative expression level of endogenous *BCL2*, *HRAS*, and *KRAS* mRNA against *β-actin* mRNA 24 h after the addition of CV measured by qRT-PCR. The qRT-PCR assay was conducted for different mRNAs extracted from three independent cell cultures in each case. The relative expression level was calculated to average these three results and normalized by the one of the results without CV. Statistical significance was evaluated using a Student’s t test. Asterisks, p < 0.05. (**B**) CD analysis showing that unfolding of *BCL2* iM by hnRNP LL in the absence (left) and presence of CV (right) at pH 6.5. (**C**) Proposed scheme of regulation of gene expression in the absence or presence of CV.

To confirm the utility of CV for *BCL2* iM in cells, we investigated intracellular gene expression from the endogenous *BCL2* gene in the MCF-7 human breast cancer cell line (see experimental section). Cancer cells were expected to have an intracellular pH indicating ~ 7.4^54^, although some i-motif formations were identified^13^. We quantified the mRNAs transcribed from *BCL2* using qRT-PCR. The expression levels of mRNA in the absence and presence of the ligand were normalised to the expression level of *β-actin* mRNA, which is a typical housekeeping gene. Interestingly, the addition of CV decreased the expression levels of *BCL2* relative to that of *β-actin* in a dose-dependent manner (Fig. 6A). Furthermore, we verified that the expression of the reference gene *β-actin* remained unaffected when using 1 and 5 μM CV in MCF-7 cells. This observation indicates that the concentration ranges of CV used in this study did not exhibit significant toxicity to the MCF-7 cell lines. These results indicate that CV repressed the transcription of the *BCL2* gene. Previously, IMC-48 that binds to *BCL2* iM upregulated the transcription of the *BCL2* gene^16^. This opposite effect might be due to the differential binding sites of the ligands to the loops of *BCL2* iM. IMC-48 was reported to bind to the central loop of the i-motif, whereas in our case, CV was found to bind to the first and third loops of *BCL2* iM. As reported by Hurley’s group, molecular recognition of the *BCL2* iM by the transcriptional factor, heterogeneous nuclear ribonucleoprotein L-like (hnRNP LL), involves the first and third loops of the i-motif for transcriptional activation^22^. For this process, the binding of RNA recognition motifs (RRMs) of hnRNP LL requires two consensus sequences found in the first (CCCGC) and third (CGCCC) loops of *BCL2* iM sequence. It has been suggested that *BCL2* iM has an equilibrium between the hairpin and i-motif structures at neutral pH. Furthermore, hnRNP LL has been shown to preferentially bind to the hairpin form of *BCL2* iM ^44^. As IMC-48 binds to the *BCL2* iM central loop, the recognition process of hnRNP LL remains unaltered, and upregulates the *BCL2* gene expression. To check whether there was a competition between hnRNP LL protein and CV in binding to *BCL2* iM, we performed CD measurements (Fig. 6B). In the absence of CV, the ellipticity of ~ 284 nm band (at pH 6.5) of *BCL2* iM gradually decreased with a blue shift in the peak position with increasing hnRNP LL concentration (up to 2 equiv of DNA), indicating partial unfolding of *BCL2* iM with the addition of hnRNP LL (Fig. 6B left). On the other hand, in the presence of CV, the reduction of the ellipticity and the blue shift by hnRNP LL were slight especially by the addition of 0.2 and 1 equiv of hnRNP LL compared with those in the absence of CV (Fig. 6B right). The peaks in the regions longer than 300 nm in the presence of CV could be induced CD signals of CV upon binding to DNA. It was further observed that CV exhibited similar interactions with the first and third loops in the *BCL2* iM, regardless of the presence of the hairpin structure. However, the complete reduction in fluorescence intensity of CV upon binding to *BCL2* iM (Fig. S3) indicates that CV does not bind to the hairpin form. Therefore, the binding of CV to *BCL2* iM interferes with the transition to the hairpin form, which is preferred by hnRNP LL. Therefore, the binding of CV to the consensus sequences of the first and third loops possibly hinders the recognition process by the transcription factor resulting in downregulation of gene expression (Fig. 6C).

To check the specificity of CV on *BCL2*, we compared the expression levels of *BCL2* with the *HRAS* and *KRAS* genes in the presence of CV. Insignificant change in the expression levels of the *HRAS* and *KRAS* genes in the presence of CV relative to the remarkable decrease in the expression level of the *BCL2* gene with CV (Fig. 6A) ensures the specific effect of CV on *BCL2*. The gene expression of *HRAS* iM did not respond due to lack of the binding sites of CV within the interloop region, which was also confirmed by the fluorescence assay (Fig. 2). Although the *KRAS* mid iM does not significantly differ in the number of bases in the first (7 nucleotides) and third (7 nucleotides) loops^17^ from *BCL2* iM (8 and 7 nucleotides in the first and third loops, respectively), it lacks guanine bases, which are proven to be essential for binding CV to *BCL2* iM, justifying the relative expression levels of the genes in the presence of CV. Furthermore, the possibility of CV binding to *BCL2* G4 in cells cannot be excluded. Our results indicate that CV considerably decreased the gene expression level of *BCL2* than those of *HRAS* and *KRAS*, complying with the relative gene expression levels of the i-motifs in the cell-based assay experiment with CV (Fig. 6A). However, reduced expression of *HRAS* and *KRAS* suggests that CV interacts with the G4s of *HRAS* and *KRAS*. Thus, compared to *KRAS* and *HRAS*, CV preferentially linked to the i-motif of the *BCL2* gene, although some downregulation of *BCL2* gene expression may be due to CV binding to *BCL2* G4. Thus, this study indicates the novel function of i-motifs for regulating gene expression that can be tuned by targeting the loop regions of i-motifs using specific small molecules.

## Conclusion

In this study, we demonstrated that the loop region of i-motif can be used as a novel platform for the specific binding of small molecules revealing light-up fluorescence properties. CV was found to be a specific binder of *BCL2* iM over other i-motifs, G4s, and non-tetraplex DNAs. It was observed to bind within the specific site created within the first and third loops of the i-motif, whereas CV did not bind to the hairpin form of the *BCL2* iM sequence. Through the local stabilisation of the structure of these loops by CV, the gene expression of *BCL2* in cancer cells was successfully repressed. Therefore, the CV-*BCL2* iM system can be a promising tool for cancer theranostics. Furthermore, the dynamic behaviour of the binding of CV to the loop regions of *BCL2* iM was regulated by crowding environment. This property would be beneficial to analyse or regulate the i-motif formation in different cancer cells, as the formation of i-motifs depends on intracellular conditions, such as cell cycles. Therefore, targeting the dynamics of loop regions to regulate i-motif formation would be a breakthrough approach to analyse and control the i-motif formations in different types of cells, including cancer cells with different malignancies.

## Methods

### Materials

All the DNA oligonucleotide sequences used in this investigation were purchased from Japan Bio Services Co., Ltd. and purified using high-performance liquid chromatography (HPLC). The DNA samples were dissolved in Milli-Q water and stored at −20 °C until use. The concentrations of the oligonucleotides were determined by measuring the absorbance at 260 nm at 90 °C using the extinction coefficients. Potassium dihydrogen phosphate (KH_2_PO_4_), potassium chloride (KCl), ethylene glycol (EG), and poly(ethylene glycol) 200 (PEG200) were purchased from Wako Pure Chemical Industries (Japan), poly(ethylene glycol) 8000 (PEG8000) and Ficoll70 were purchased from Sigma-Aldrich (USA) and dipotassium ethylenediaminetetraacetate (K_2_EDTA) was purchased from Dojindo Molecular Technologies (Japan), and all these chemicals were used as received. BBR, CV, ThT, and TO were purchased from Sigma-Aldrich (USA); PR and BG were purchased from Wako Pure Chemical Industries (Japan); MG and NR were purchased from TCI (Japan), and all these dye molecules were used without further purification. Stock solutions (2 mM) of the dyes were prepared in Milli-Q water and the required aliquots were taken from the stock solutions for experiments. Addition of the dye solutions to the buffer did not change the pH of the solutions during all experiments.

### Protein expression and purification

The gene of truncated hnRNP LL was constructed for expression as a C-terminal his-tagged protein in *E. coli* by Eurofins Genomic Service (Tokyo, Japan). The truncated genes were subcloned into pET-21a (Novagen). The resulting plasmid was transfected into *E. coli* BL21(DE3). Transformants were cultured in the Overnight Express medium (Merck) including ampicillin at 37 °C for 20 h. The cells were harvested and lysed by sonication. His-tagged hnRNP LL was purified from the soluble fraction of the extract using His-trap HP (Cytiva) by using buffer containing 20 mM Tris–HCl (pH 7.2) and 500 mM NaCl with a gradient of imidazole. The purified fractions were further purified on a HiTrap Heparin HP column (Cytiva). His-tagged hnRNP LL was eluted with a gradient of NaCl. Fractions were concentrated by Amicon Ultra (Merck) and stored at 4 °C until use.

### UV melting studies

UV melting profiles were obtained using a Shimadzu UV-1700 spectrophotometer with a thermoprogrammer. All the experiments were conducted in a buffer containing 10 mM KH_2_PO_4_, 1 mM K_2_EDTA, and 50 mM KCl with pH 5.0 at 25 °C. EG, PEG, and Ficoll were added to the buffer as required. The sample solutions were kept at 95 °C for 5 min, followed by a decrease in temperature to 0 °C at a rate of 0.5 °C min^−1^ to anneal. Thereafter, the samples were heated to 95 °C at a rate of 0.5 °C min^−1^ to melt the DNA motif. A constant stream of dry N_2_ gas was flushed to avoid water condensation on the exterior of the cuvette. The melting profiles were analysed by a fitting equation considering the two-state melting process including subtraction of temperature dependent baselines for the coil and helix forms^55^. No information was lost during the analysis of the normalised melting profiles when compared with analyses of the original ones. *T*_m_ values were determined from the ∆*H*° and ∆*S*° values of the melting profiles. The experiments were performed in triplicate.

### Fluorescence studies

For scanning the fluorescence response of the dyes in the presence of DNA, 15 µM DNA was taken in a buffer solution containing 10 mM KH_2_PO_4_, 1 mM K_2_EDTA, and 50 mM KCl (pH 5.0 at 25 °C). EG, PEG, and Ficoll were added to the buffer as required. The DNA solutions were heated at 95 °C for 3 min, and then cooled down to 25 °C at the rate of −1.0 °C min^−1^ and incubated for 30 min. After this, 6 µM dye solution was added to each DNA solution. Then the fluorescence response was recorded on a Varioskan Flash microplate reader (Thermo Fisher Scientific, USA). The excitation and emission monitoring wavelengths for the dyes were CV: 580 nm and 620 nm, MG: 620 nm and 690 nm, BG: 610 nm and 670 nm, PR: 550 nm and 620 nm, NR: 535 and 610 nm, BBR: 350 nm and 530 nm, ThT: 425 nm and 490 nm, and TO: 488 nm and 535 nm. The experiments were performed in triplicate. For the dye-DNA binding constant calculation, the fluorescence data was fitted with a 1:1 non-linear curve-fitting equation^33, 56^:

$$\mathrm{F}=\mathrm{Fmin}+ \frac{\mathrm{Fmax}-\mathrm{Fmin}}{2[\mathrm{DNA}]}\times {\left(\left[\mathrm{DNA}\right]+\left[\mathrm{Dye}\right]+1/\mathrm{K}\right)-{(\left(\left[\mathrm{DNA}\right]+\left[\mathrm{Dye}\right]+1/\mathrm{K}\right)}^{2}-4[DNA][Dye])}^{0.5}$$

where F is the fluorescence intensity of the dye in the presence of DNA, F_min_ is the fluorescence intensity of the dye in the absence of DNA, and F_max_ is the maximum fluorescence intensity after titration with DNA. K is the dye-DNA binding constant.

### Job’s plot

To determine the stoichiometry between CV and *BCL2* iM, independent fluorescence intensities were obtained using various concentrations of CV and *BCL2* iM, while the total concentrations of CV and *BCL2* iM were fixed at 10 μM. The experiments were performed in triplicate.

### Circular dichroism (CD) measurements

The CD spectra were measured on a JASCO J-1500 spectropolarimeter equipped with a temperature controller. All spectra were collected at 4 °C. A stream of dry N_2_ gas was constantly flushed in the cuvette-holding chamber to avoid water condensation on the cuvette exterior. The spectra were measured in the range of 200 to 350 nm in 0.1 cm path-length cuvettes, with a scan rate of 100 nm min^−1^. The composition of buffer was 10 mM KH_2_PO_4_, 1 mM K_2_EDTA, and 50 mM KCl (pH 5.0 at 25 °C).

For the hnRNP LL assay, 5 μM *BCL2* iM was incubated with or without 50 µM CV and different concentration of hnRNP LL (0, 0.2, 1 and 2 equiv.) in 50 mM MES-KOH (pH 6.5), 100 mM KCl and 1 mM DTT at 25 °C for 5 min before CD spectra measurement. To correct baseline, CD spectra of *BCL2* iM containing hnRNP LL with or without CV were subtracted by the spectra of the solution containing each concentration of hnRNP LL with or without CV in the buffer.

### Molecular dynamics (MD) simulation

The structures of the *BCL2* iM and its mutant sequences were prepared by modification of NMR structure for the i-motif DNA structure (PDB ID: 1EL2)^48^. The structures of these sequences were obtained by base substitution and DNA strand extension using Discovery Studio Visualizer^57^. The the simulation box size was set to ensure a minimum distance of 20 Å from the DNA to the wall of the box. The box was filled up by water molecules, potassium and chloride ions as a 0.05 M concentration of KCl solution. The optimisations by the steepest descent method were performed with 10,000 steps for solvent and 10,000 steps for the whole systems. The systems were gradually heated from 0 to 298.15 K for 100 ps and equilibrated by the constant condition of the particle number, volume, and temperature (NVT ensemble) for 1 ns. Equilibrations by the constant condition of the particle number, pressure, and temperature (NPT ensemble) was performed at 1 atm and 298.15 K for 1 ns. Finally, sampling simulations by the NPT ensemble were performed for 100 ns. Three of these simulations with random initial velocity vectors were run for each system using GROMACS Ver. 2020.6. Force fields of ff14SB^58^ and TIP3P^59^ employed general nucleotides and water molecules. The force field of protonated cytosine nucleotides was GAFF^60^ with atom charges determined by the RESP calculation^61^ of Gaussian09^62^.

### Cell-based assay experiments

Human breast cancer cell line MCF-7 (ECACC 86012803) was purchased from the European Collection of Authenticated Cell Cultures (ECACC). Cells were cultured in Dulbecco's modified Eagle's medium supplemented with 10% fetal bovine serum in the absence and presence of ligand at concentrations of 1.0 and 5.0 µM; after 24 h, the cells were collected for extracting endogenous RNA. Total RNA was extracted using the Qiagen RNeasy Mini kit and cDNA was prepared according to the manufacturer’s protocol. Quantitative reverse transcription qRT-PCR analysis was performed using SYBR^®^ Green Real-time PCR Master Mix (Toyobo) using the StepOne™ Real-Time PCR System (Applied Biosystems). The transcription conditions of *BCL2*, *HRAS*, *KRAS* and *β-actin* genes were: 60 s at 95 °C, 40 cycles at 95 °C for 15 s, and 55 °C for 60 s. The primer sets were as follows: *bcl2* forward, 5′-ATCGCCCTGTGGATGACTGAGT-3′, reverse 5′-GCCAGGAGAAATCAAACAGAGGC-3′; *hras* forward, 5′-ACGCACTGTGGAATCTCGGCAG-3′, reverse 5′-TCACGCACCAACGTGTAGAAGG-3′; *kras* forward, 5′-CAGTAGACACAAAACAGGCTCAG-3′, reverse 5′-TGTCGGATCTCCCTCACCAATG-3′; *β-actin* forward, 5′-TTTAATAGTCATTCCAAATATGAGA-3′, reverse 5′-ACATAATTTACACGAAAGCA-3′. *β-actin* was used as a control for data normalisation. Experiments were performed in triplicate for three independent cultures of cells. The relative expression level was calculated to average these three results and normalized by the one of the results without CV. Statistical significance was evaluated using a Student’s t test, and results were considered statistically significant when P < 0.05.

### Supplementary Information

Supplementary Information.

## Data Availability

The datasets used and/or analysed during the current study are available from the corresponding author on reasonable request.
