# Supplementary material for: Theranostic approach to specifically targeting the interloop region of BCL2 i-motif DNA by crystal violet
Source: Sci Rep. 2023 Sep 1;13:14338. doi: 10.1038/s41598-023-39407-9 (PMC10474294; doi:10.1038/s41598-023-39407-9)
Supplement: Supplementary file 1 — Supplementary Information. [file 41598_2023_39407_MOESM1_ESM.pdf]

Supporting information

Theranostic approach to specifically targeting the interloop region of *BCL2* i-motif DNA by crystal violet

Sinjan Das,[1,a] Shuntaro Takahashi,[1,a] Tatsuya Ohyama,[1] Sudipta Bhowmik,[2,3] and Naoki Sugimoto*[1,4]

[1]Frontier Institute for Biomolecular Engineering Research (FIBER), Konan University, 7-1-20 Minatojima-Minamimachi, Kobe 650-0047, Japan.

[2]Department of Biophysics, Molecular Biology and Bioinformatics, University of Calcutta, 92, A.P.C Road, Kolkata-700009, India.

[3]Mahatma Gandhi Medical Advanced Research Institute (MGMARI), Sri Balaji Vidyapeeth (Deemed to be University), Pondy-Cuddalore Main Road, Pillayarkuppam, Pondicherry, 607402, India.

[4]Graduate School of Frontiers of Innovative Research in Science and Technology (FIRST), Konan University, 7-1-20 Minatojima-Minamimachi, Kobe 650-0047, Japan.

* To whom correspondence should be addressed. Tel: (+81)78-303-1457; Fax: (+81)78-303-1495; Email: sugimoto@konan-u.ac.jp

[a]The authors equally contributed to this manuscript.

Table S1. Abasic and mutated *BCL2* iM DNA sequences used in this study (5′ → 3′)

BCL2 iM 5′L-A[a]	CAGCCCCGCT**<u>XXXXX</u>**CCCCTTCCTCCCGCGCCCGCCCCT
BCL2 iM CL-A[a]	CAGCCCCGCTCCCGCCCCC**<u>XXXXX</u>**CCCGCGCCCGCCCCT
BCL2 iM 3′L-A[a]	CAGCCCCGCTCCCGCCCCCTTCCTCCCG**<u>XXXXX</u>**GCCCCT
5′L-CTCAT	CAGCCCCGCT**<u>CTCAT</u>**CCCCTTCCTCCCGCGCCCGCCCCT
5′L-CCCGT	CAGCCCCGCT**<u>CCCGT</u>**CCCCTTCCTCCCGCGCCCGCCCCT
5′L-TCCAT	CAGCCCCGCT**<u>TCCAT</u>**CCCCTTCCTCCCGCGCCCGCCCCT
5′L-TTCAT	CAGCCCCGCT**<u>TTCAT</u>**CCCCTTCCTCCCGCGCCCGCCCCT
5′L-CCCAC	CAGCCCCGCT**<u>CCCAC</u>**CCCCTTCCTCCCGCGCCCGCCCCT
5′L-CCTAT	CAGCCCCGCT**<u>CCTAT</u>**CCCCTTCCTCCCGCGCCCGCCCCT
5′L-TTCGT	CAGCCCCGCT**<u>TTCGT</u>**CCCCTTCCTCCCGCGCCCGCCCCT
5′L-TTCAC	CAGCCCCGCT**<u>TTCAC</u>**CCCCTTCCTCCCGCGCCCGCCCCT
5′L-TCCGT	CAGCCCCGCT**<u>TCCGT</u>**CCCCTTCCTCCCGCGCCCGCCCCT
5′L-TCTAT	CAGCCCCGCT**<u>TCTAT</u>**CCCCTTCCTCCCGCGCCCGCCCCT
5′L-TCCAC	CAGCCCCGCT**<u>TCCAC</u>**CCCCTTCCTCCCGCGCCCGCCCCT
5′L-CTTGT	CAGCCCCGCT**<u>CTTGT</u>**CCCCTTCCTCCCGCGCCCGCCCCT
5′L-TCTAC	CAGCCCCGCT**<u>TCTAC</u>**CCCCTTCCTCCCGCGCCCGCCCCT
5′L-CTTAC	CAGCCCCGCT**<u>CTTAC</u>**CCCCTTCCTCCCGCGCCCGCCCCT
5′L-TTTAC	CAGCCCCGCT**<u>TTTAC</u>**CCCCTTCCTCCCGCGCCCGCCCCT
5′L-TTTGT	CAGCCCCGCT**<u>TTTGT</u>**CCCCTTCCTCCCGCGCCCGCCCCT
5′L-TTTAT	CAGCCCCGCT**<u>TTTAT</u>**CCCCTTCCTCCCGCGCCCGCCCCT
5′L-TCTGT	CAGCCCCGCT**<u>TCTGT</u>**CCCCTTCCTCCCGCGCCCGCCCCT
5′L-CTCGC	CAGCCCCGCT**<u>CTCGC</u>**CCCCTTCCTCCCGCGCCCGCCCCT
5′L-TCCGC	CAGCCCCGCT**<u>TCCGC</u>**CCCCTTCCTCCCGCGCCCGCCCCT
5′L-TTCGC	CAGCCCCGCT**<u>TTCGC</u>**CCCCTTCCTCCCGCGCCCGCCCCT
5′L-CCTGC	CAGCCCCGCT**<u>CCTGC</u>**CCCCTTCCTCCCGCGCCCGCCCCT
5′L-TCTGC	CAGCCCCGCT**<u>TCTGC</u>**CCCCTTCCTCCCGCGCCCGCCCCT
5′L-TTTGC	CAGCCCCGCT**<u>TTTGC</u>**CCCCTTCCTCCCGCGCCCGCCCCT
3′L-CACTC	CAGCCCCGCTCCCGCCCCCTTCCTCCCG**<u>CACTC</u>**GCCCCT
3′L-TACTC	CAGCCCCGCTCCCGCCCCCTTCCTCCCG**<u>TACTC</u>**GCCCCT
3′L-TACTT	CAGCCCCGCTCCCGCCCCCTTCCTCCCG**<u>TACTT</u>**GCCCCT
3′L-CACCC	CAGCCCCGCTCCCGCCCCCTTCCTCCCG**<u>CACCC</u>**GCCCCT
3′L-TATTC	CAGCCCCGCTCCCGCCCCCTTCCTCCCG**<u>TATTC</u>**GCCCCT
3′L-TACCC	CAGCCCCGCTCCCGCCCCCTTCCTCCCG**<u>TACCC</u>**GCCCCT

3′L-CGTTC	CAGCCCCGCTCCCGCCCCCTTCCTCCCG**<u>CGTTC</u>**GCCCCT
3′L-CACTT	CAGCCCCGCTCCCGCCCCCTTCCTCCCG**<u>CACTT</u>**GCCCCT
3′L-TGTTC	CAGCCCCGCTCCCGCCCCCTTCCTCCCG**<u>TGTTC</u>**GCCCCT
3′L-CATTC	CAGCCCCGCTCCCGCCCCCTTCCTCCCG**<u>CATTC</u>**GCCCCT
3′L-TGTTT	CAGCCCCGCTCCCGCCCCCTTCCTCCCG**<u>TGTTT</u>**GCCCCT
3′L-CGTTT	CAGCCCCGCTCCCGCCCCCTTCCTCCCG**<u>CGTTT</u>**GCCCCT
3′L-TGCTT	CAGCCCCGCTCCCGCCCCCTTCCTCCCG**<u>TGCTT</u>**GCCCCT
3′L-CATCC	CAGCCCCGCTCCCGCCCCCTTCCTCCCG**<u>CATCC</u>**GCCCCT
3′L-TATCC	CAGCCCCGCTCCCGCCCCCTTCCTCCCG**<u>TATCC</u>**GCCCCT
3′L-CACCT	CAGCCCCGCTCCCGCCCCCTTCCTCCCG**<u>CACCT</u>**GCCCCT
3′L-CGTCC	CAGCCCCGCTCCCGCCCCCTTCCTCCCG**<u>CGTCC</u>**GCCCCT
3′L-TACCT	CAGCCCCGCTCCCGCCCCCTTCCTCCCG**<u>TACCT</u>**GCCCCT
3′L-CGCTC	CAGCCCCGCTCCCGCCCCCTTCCTCCCG**<u>CGCTC</u>**GCCCCT
3′L-TGCCC	CAGCCCCGCTCCCGCCCCCTTCCTCCCG**<u>TGCCC</u>**GCCCCT
3′L-TATCT	CAGCCCCGCTCCCGCCCCCTTCCTCCCG**<u>TATCT</u>**GCCCCT
3′L-TGCCT	CAGCCCCGCTCCCGCCCCCTTCCTCCCG**<u>TGCCT</u>**GCCCCT
3′L-TGTCC	CAGCCCCGCTCCCGCCCCCTTCCTCCCG**<u>TGTCC</u>**GCCCCT
3′L-CATCT	CAGCCCCGCTCCCGCCCCCTTCCTCCCG**<u>CATCT</u>**GCCCCT
3′L-CGTCT	CAGCCCCGCTCCCGCCCCCTTCCTCCCG**<u>CGTCT</u>**GCCCCT
3′L-CGCCT	CAGCCCCGCTCCCGCCCCCTTCCTCCCG**<u>CGCCT</u>**GCCCCT
3′L-TGCTC	CAGCCCCGCTCCCGCCCCCTTCCTCCCG**<u>TGCTC</u>**GCCCCT
BCL2 2323	CAGCCCGCTCCCGCCCCTTCCTCCGCGCCCGCCCT
BCL2 4545	CAGCCCCCGCTCCCGCCCCCCTTCCTCCCCGCGCCCGCCCCCT
BCL2 5656	CAGCCCCCCGCTCCCGCCCCCCCTTCCTCCCCCGCGCCCGCCCCCCT

[a]"X" in the sequences represents the abasic sites introduced by dSpacer phosphoramidite (Glen Research). Abasic sites and mutated bases are made bold and underlined

Table S2. Thermodynamic parameters and melting temperatures of *BCL2* iM in different crowding conditions

Solution	$-\Delta H^{\circ}$ (kcal mol^{-1})	$-T\Delta S^{\circ}$ (kcal mol^{-1})	$-\Delta G^{\circ}_{37}$ (kcal mol^{-1})	T_m (°C)
10 wt% EG	74.2 ± 1.2	68.3 ± 1.4	5.9 ± 0.2	63.8 ± 0.1
10 wt% PEG200	73.2 ± 1.4	67.0 ± 1.4	6.2 ± 0.3	65.7 ± 0.3
10 wt% PEG8000	61.5 ± 1.5	55.3 ± 1.5	6.2 ± 0.3	71.8 ± 0.2
10 wt% Ficoll70	67.4 ± 1.3	61.3 ± 1.4	6.1 ± 0.2	67.9 ± 0.2

Buffer contained 10 mM KH_2PO_4, 1 mM K_2EDTA and 50 mM KCl. pH of the solution was maintained at 5.0 at 25 °C.

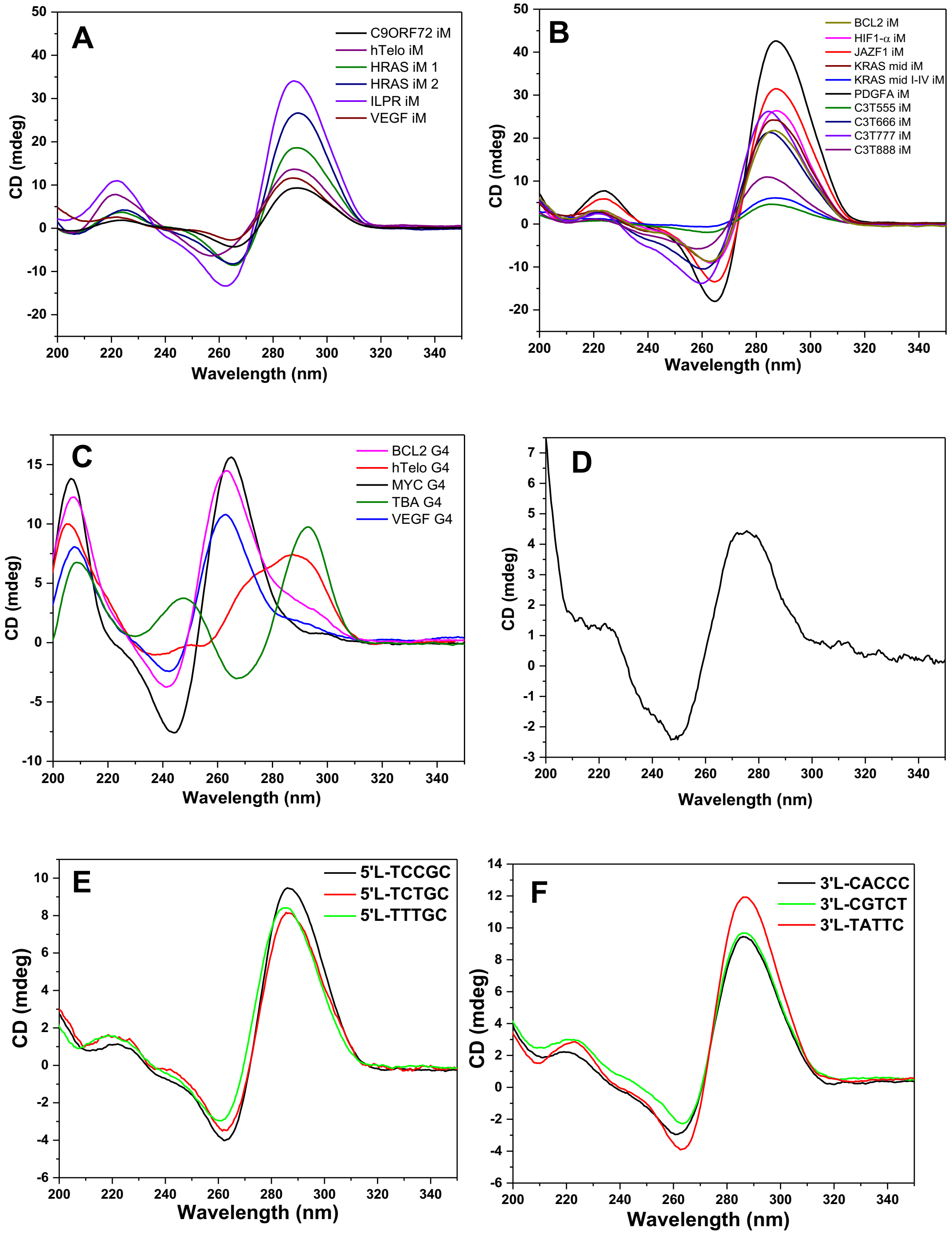

Figure S1. CD spectra of 15 μM of (A) class I i-motif, (B) class II i-motif, (C) G4 and (D) hairpin sequences used in this study. Some representative CD spectra of 5 μM of 5′ (E) and 3′ (F) loop mutants of *BCL2* iM. All measurements were carried out in 10 mM KH_2PO_4, 1 mM K_2EDTA and 50 mM KCl at pH 5.0 at 25 °C.

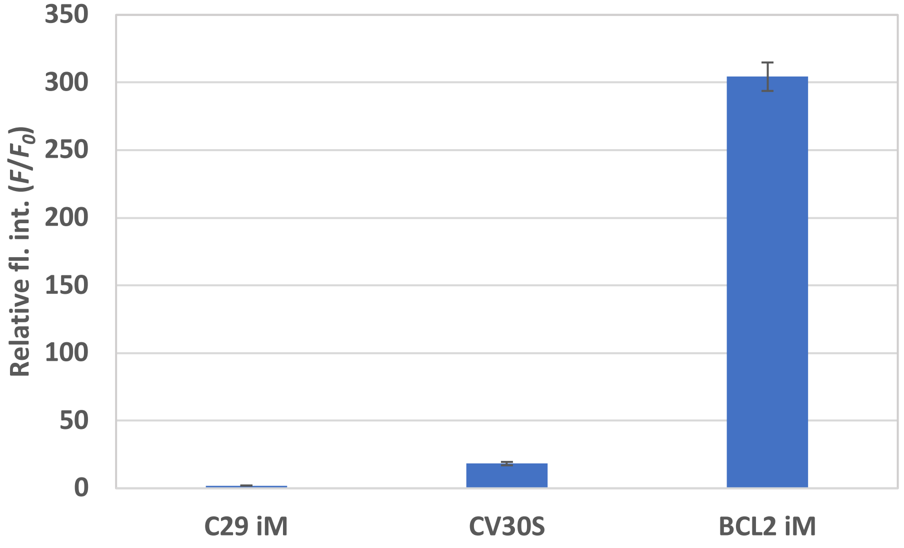

Figure S2. Fluorescence response of CV with C29 iM, CV30S and *BCL2* iM at pH 5.0 at 25 °C. The data was relative to no DNA as a control. The concentration of CV and DNAs were 6 µM and 15 µM, respectively.

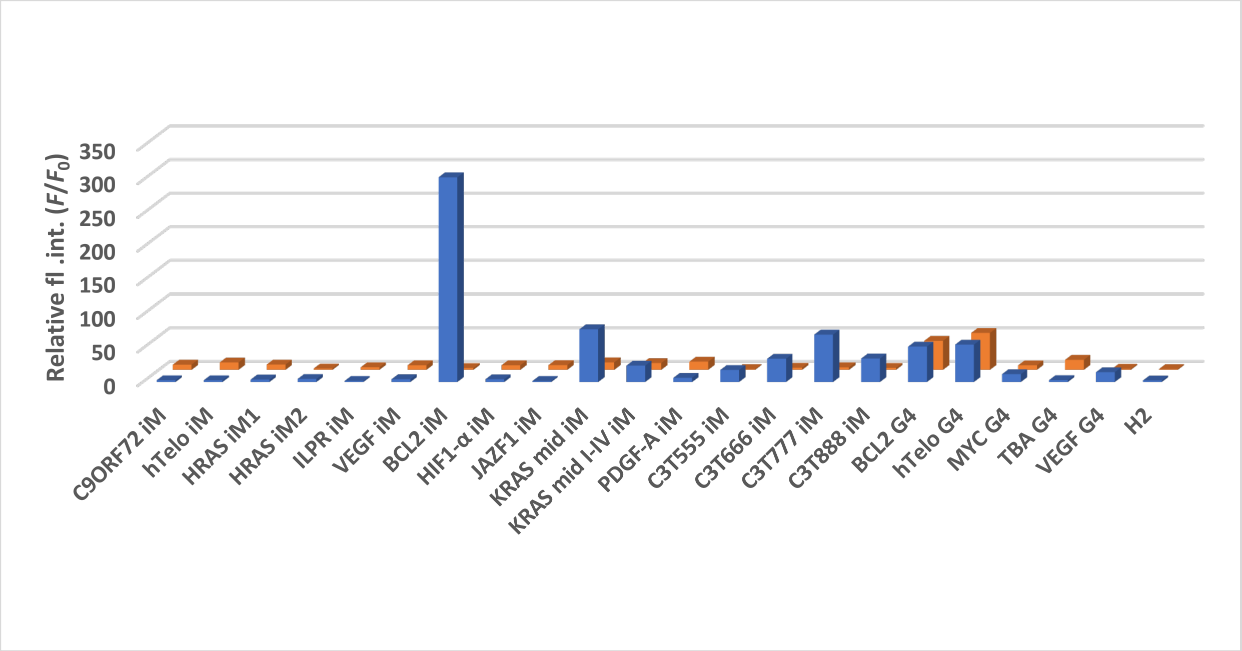

Figure S3. Fluorescence responses of CV at pH 5.0 (blue) and pH 7.0 (orange) with different DNA motifs at 25 °C. The data was relative to no DNA as a control. The concentration of CV and DNAs were 6 µM and 15 µM, respectively. The errors were within 5%.

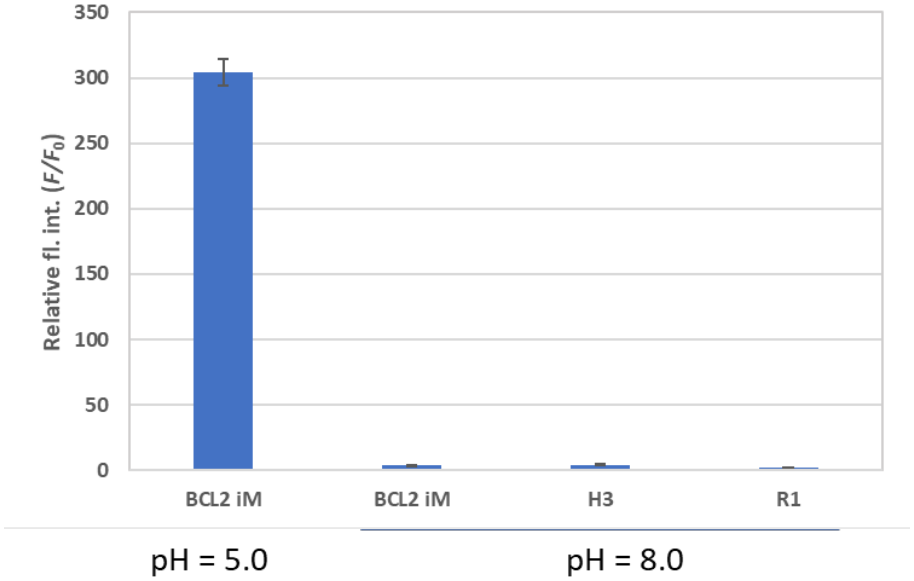

Figure S4. Fluorescence responses of CV with *BCL2* iM in 10 mM KH_2PO_4, 1 mM K_2EDTA and 50 mM KCl at pH 5.0 and the same with *BCL2* iM, H3, and R1 as C-rich, G-rich and randomly composed DNAs, respectively in 10 mM Tris-HCl, 50 mM LiCl at pH 8.0 at 25 °C. The data was relative to no DNA as the control. The concentration of CV and DNAs were 6 μM and 15 μM, respectively.

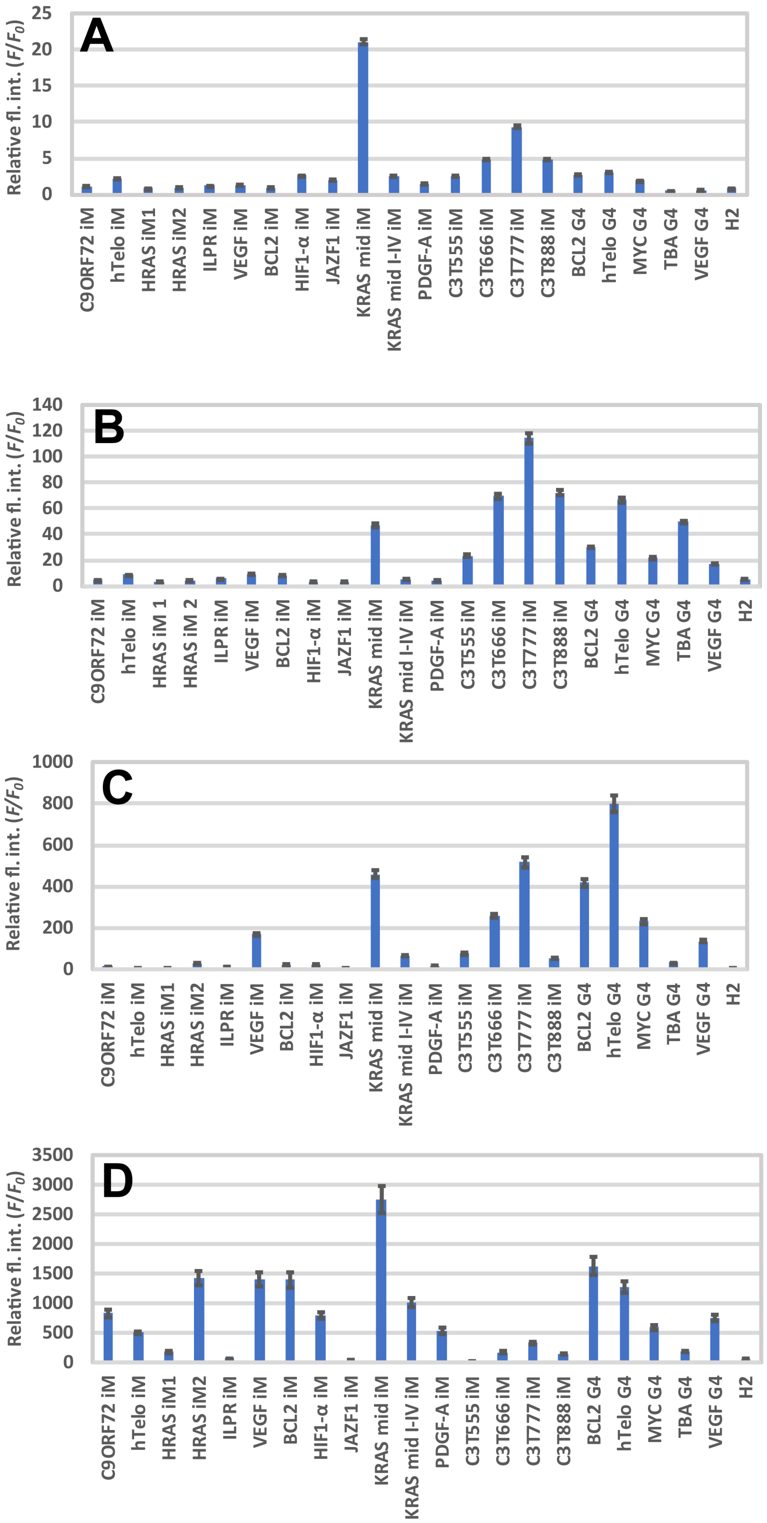

Figure S5. Fluorescence responses of (A) NR, (B) BBR, (C) ThT and (D) TO with different DNA motifs at pH 5.0 at 25 °C. The data was relative to no DNA as a control. The concentration of each ligand and DNAs were 6 µM and 15 µM, respectively.

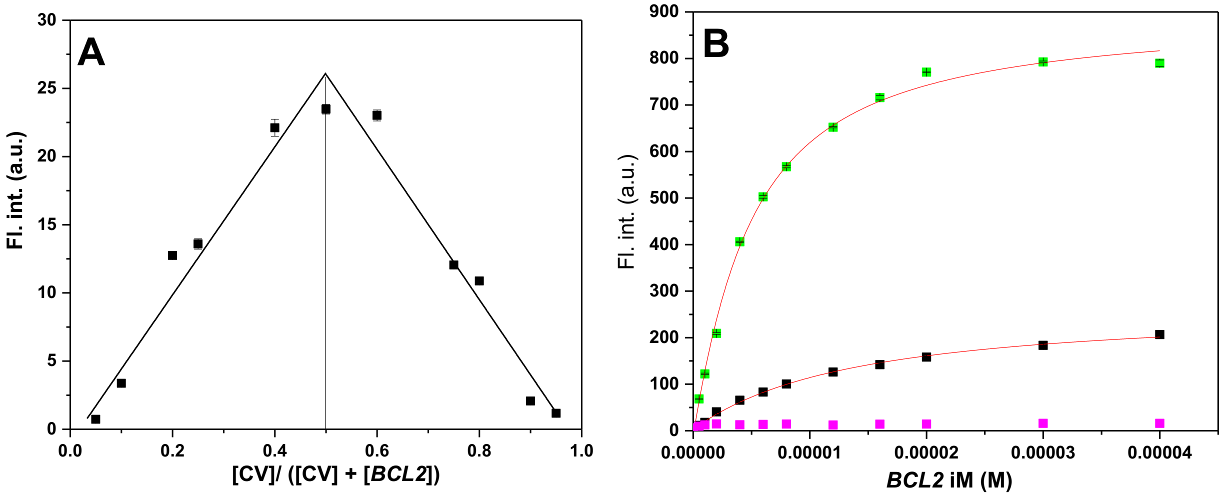

Figure S6. (A) Job's plot for CV with *BCL2* iM. (B) Fluorescence titration of 2 μM CV with varying concentration of WT *BCL2* iM (black), 5′L-TTTGC mutant (green) and 5′L-CTCAT mutant (magenta). Fitted lines were drawn in red. No fitted line could be drawn for the fluorescence data of 5′L-CTCAT mutant due to its low affinity with CV. All measurements were carried out in 10 mM KH_2PO_4, 1 mM K_2EDTA and 50 mM KCl at pH 5.0 at 25 °C.

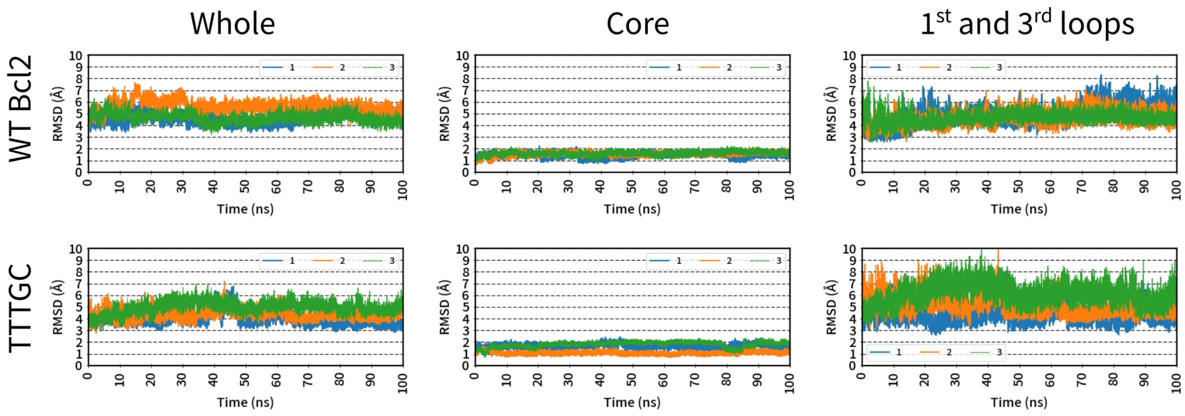

Figure S7. Change in root mean square deviation (RMSD) values for heavy atoms in *BCL2* iM in the whole structure, core region, and first and third loops. The colours indicate the different simulation runs.

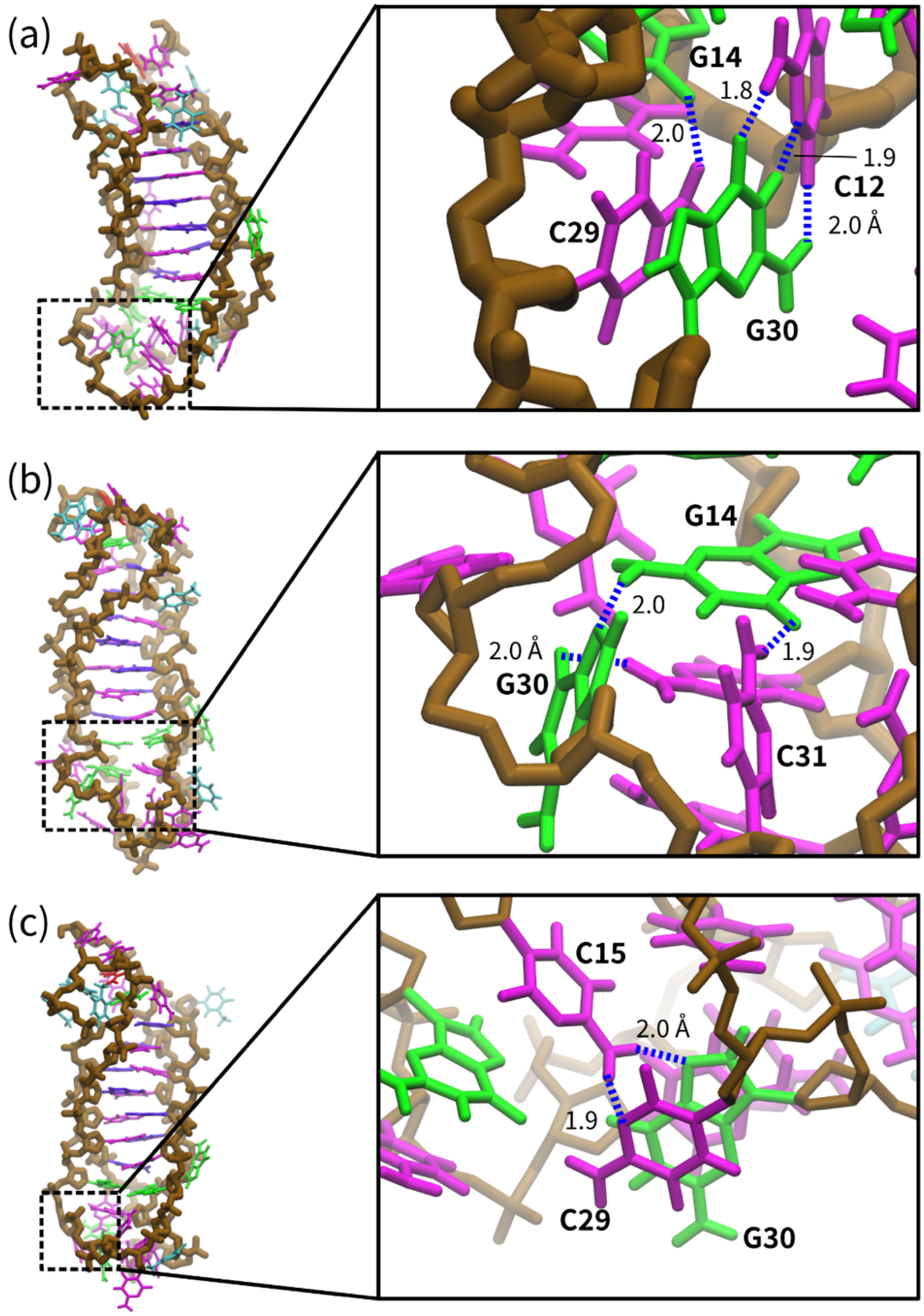

Figure S8. Representative structures of *BCL2* iM in representative snapshots of MD simulations in different three runs (a-c). The right panels are the enlarged view of the loop-loop interactions. Representation of green, magenta, purple, cyan, and brown sticks show guanine, cytosine, protonated cytosine, and thymine bases, and backbone, respectively. Blue lines show hydrogen bonds.

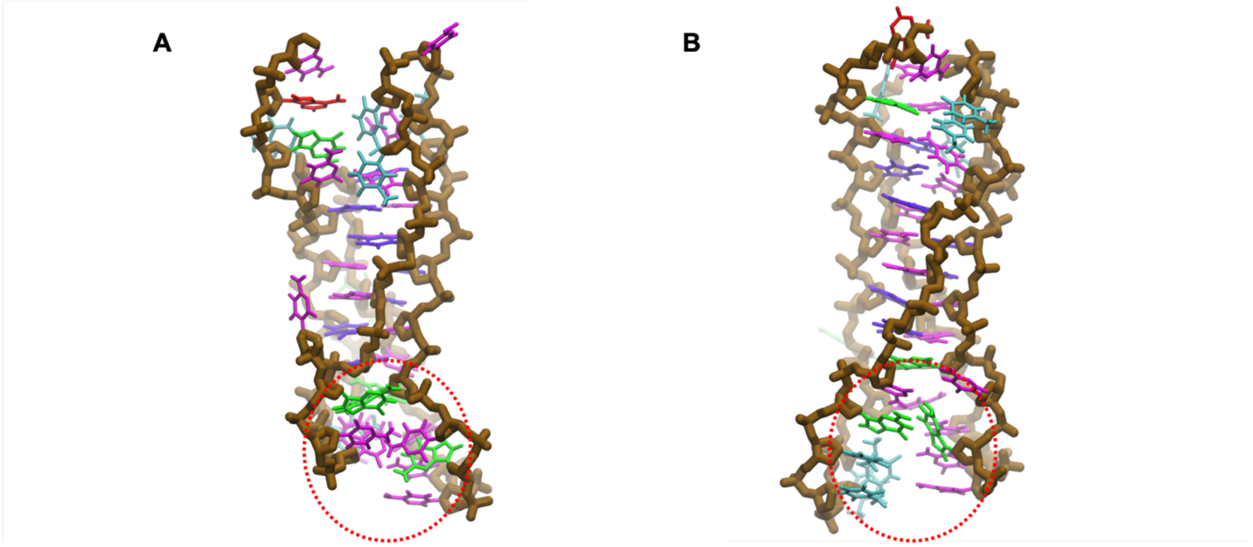

Figure S9. Representative structures of (A) WT *BCL2* iM and (B) *BCL2* iM with TTTGC mutation at the first loop in the MD simulation. The structures around first and third loops were highlighted in red circles.

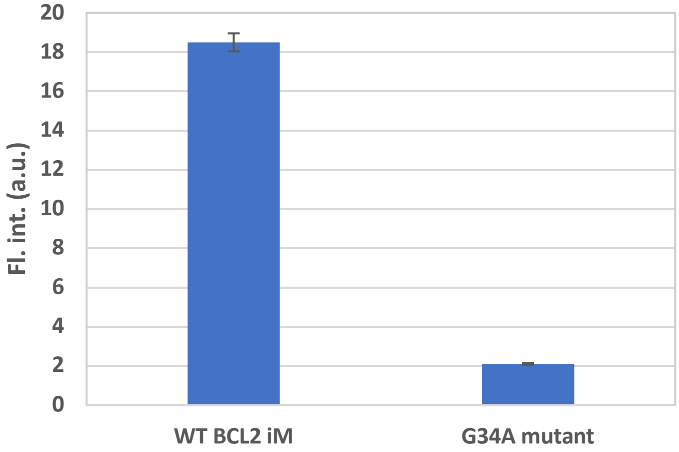

Figure S10. Fluorescence responses of 6 μM CV with 15 μM WT *BCL2* iM and its G34A mutant at pH 5.0 at 25 °C.

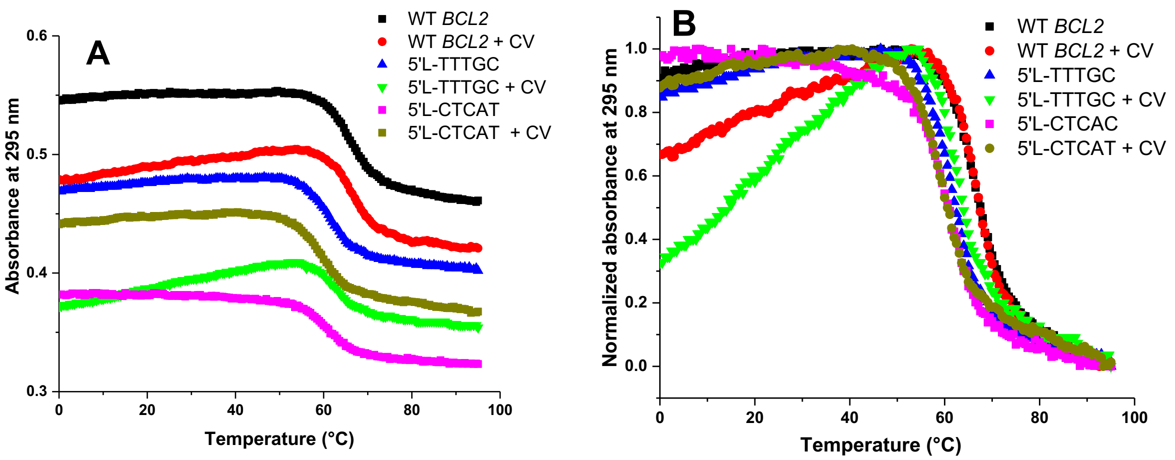

Figure S11. (A) Original and (B) normalized UV melting profiles of 5 μM WT *BCL2* iM, its 5′L-TTTGC and 5′L-CTCAT mutant in absence (black, blue and magenta) and presence of 15 μM CV (red, green and dark yellow). The buffer contained 10 mM KH_2PO_4, 1 mM K_2EDTA and 50 mM KCl at pH 5.0.

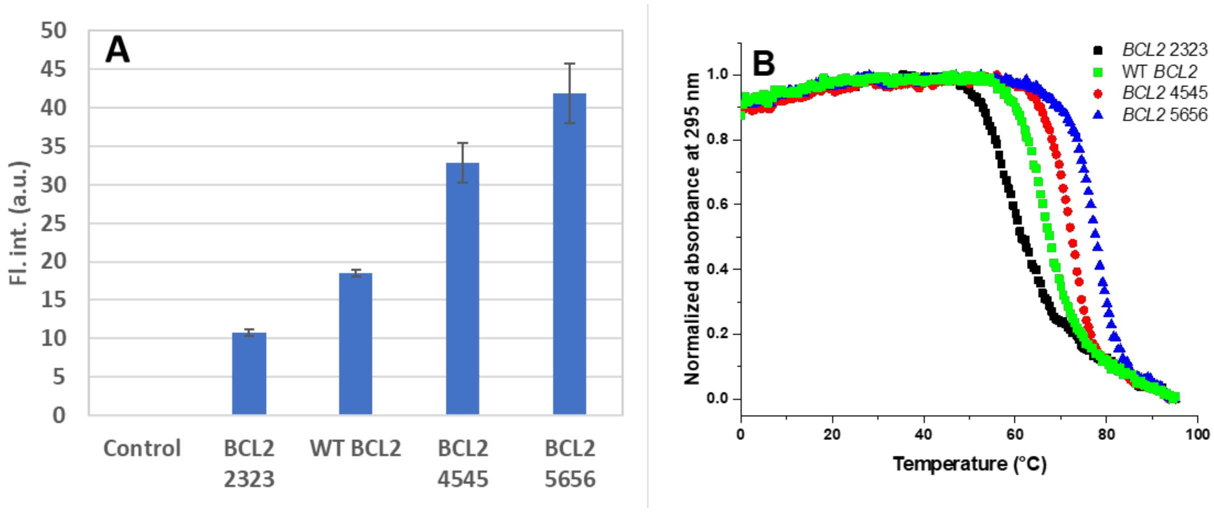

Figure S12. (A) Fluorescence responses of CV at pH 5.0 at 25 °C with varying number of C-tracts of *BCL2* iM; *BCL2* 2323 ($C_2C_3C_2C_3$ tracts), WT *BCL2* ($C_3C_4C_3C_4$ tracts), *BCL2* 4545 ($C_4C_5C_4C_5$ tracts) and *BCL2* 5656 ($C_5C_6C_5C_6$ tracts), respectively and (B) UV-melting profiles of these sequences without CV. The colors black, green, red and blue represent the melting profiles of *BCL2* 2323, WT *BCL2*, *BCL2* 4545 and *BCL2* 5656, respectively. The buffer contained 10 mM KH_2PO_4, 1 mM K_2EDTA and 50 mM KCl at pH 5.0.

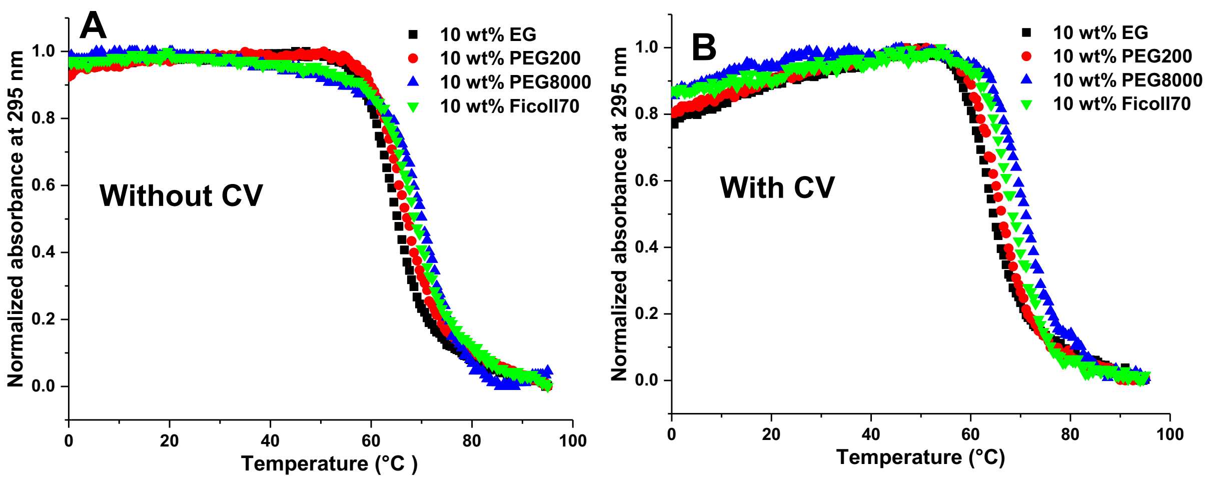

Figure S13. UV-melting profiles of 5 μM *BCL2* iM in the absence (A) and presence (B) of 15 μM CV under different crowding conditions. Black, red, blue, and green data points represent 10 wt% EG, PEG200, PEG8000 and Ficoll70, respectively. The buffer contained 10 mM KH_2PO_4, 1 mM K_2EDTA and 50 mM KCl at pH 5.0.
